# Relationship of leaf oxygen and carbon isotopic composition with transpiration efficiency in the C_4_ grasses *Setaria viridis* and *Setaria italica*

**DOI:** 10.1093/jxb/erx185

**Published:** 2017-07-11

**Authors:** Patrick Z Ellsworth, Patrícia V Ellsworth, Asaph B Cousins

**Affiliations:** School of Biological Sciences, Washington State University, Pullman, WA, USA

**Keywords:** C4 photosynthesis, carbon isotopic composition, drought, gas exchange, oxygen isotopic composition, Setaria italica, Setaria viridis, stable isotopes, transpiration efficiency, water limitation

## Abstract

Leaf carbon and oxygen isotope ratios can potentially provide a time-integrated proxy for stomatal conductance (*g*_s_) and transpiration rate (*E*), and can be used to estimate transpiration efficiency (TE). In this study, we found significant relationships of bulk leaf carbon isotopic signature (δ^13^C_BL_) and bulk leaf oxygen enrichment above source water (Δ^18^O_BL_) with gas exchange and TE in the model C_4_ grasses *Setaria viridis* and *S. italica*. Leaf δ^13^C had strong relationships with *E*, *g*_s_, water use, biomass, and TE. Additionally, the consistent difference in δ^13^C_BL_ between well-watered and water-limited plants suggests that δ^13^C_BL_ is effective in separating C_4_ plants with different availability of water. Alternatively, the use of Δ^18^O_BL_ as a proxy for *E* and TE in S. *viridis* and *S. italica* was problematic. First, the oxygen isotopic composition of source water, used to calculate leaf water enrichment (Δ^18^O_LW_), was variable with time and differed across water treatments. Second, water limitations changed leaf size and masked the relationship of Δ^18^O_LW_ and Δ^18^O_BL_ with *E*. Therefore, the data collected here suggest that δ^13^C_BL_ but not Δ^18^O_BL_ may be an effective proxy for TE in C_4_ grasses.

## Teaser

Leaf carbon isotopic composition correlated well with transpiration efficiency, while leaf oxygen isotopic composition was confounded by leaf length in the C_4_ grasses *Setaria viridis* and *Setaria italica*.

## Introduction

The bulk leaf carbon isotopic signature (δ^13^C_BL_) can potentially provide time-integrated proxies of stomatal conductance and transpiration efficiency (TE), where TE is defined as the quantity of carbon fixed per unit water lost through transpiration (for a glossary of terms, see Table 1). For example, δ^13^C_BL_ has been successfully used in wheat breeding programs to screen for TE (Farquhar and Richards, 1984; Cabrera-Bosquet *et al.*, 2009*a*, 2011; Yousfi *et al.*, 2012; Araus *et al.*, 2013) and has been studied in C_4_ species such as maize (Monneveux *et al.*, 2007; Cabrera-Bosquet *et al.*, 2009*b*,*c*), sorghum (Henderson *et al.*, 1998), sugarcane (Saliendra *et al.*, 1996), and pearl millet (Brück *et al.*, 2000). Additionally, bulk leaf oxygen enrichment above source water (S) (Δ^18^O_BL_=δ^18^O_BL_−δ^18^O_S_) has been proposed as a proxy for transpiration rate (*E*) when comparing plants grown together under the same atmospheric and climatic conditions (Barbour *et al.*, 2000*a*; Condon *et al.*, 2004; Barbour, 2007). For example, Δ^18^O_BL_ has been shown to vary with *E* in several crop species such as tea (Sheshshayee *et al.*, 2010), sunflower (Sheshshayee *et al.*, 2005), cowpea (Bindu Madhava *et al.*, 1999), and wheat (Cabrera-Bosquet *et al.*, 2009*a*). However, to date C_4_ plant-breeding programs have not generally used stable isotopes to phenotype or select for TE.

**Table 1.** Glossary of terms

Term	Definition
Δ^13^C	Photosynthetic carbon discrimination (δ^13^C_ambient_−δ^13^C_BL_)
δ^13^C_BL_	Leaf carbon isotopic composition
δ^18^O_LW_	Oxygen isotopic composition of leaf water
δ^18^O_SW_	Oxygen isotopic composition of soil water
δ^18^O_RC_	Oxygen isotopic composition of root crown water
δ^18^O_S_	Oxygen isotopic composition of source water
δ^18^O_I_	Oxygen isotopic composition of irrigation water
Δ^18^O_LW_	Leaf water enrichment (δ^18^O_LW_−δ^18^O_S_)
Δ^18^O_BL_	Bulk leaf enrichment (δ^18^O_BL_−δ^18^O_S_)
Δ^18^O_v_	Enrichment of water vapor above source water (δ^18^O_v_−δ^18^O_S_)
*C*_i_/*C*_a_	Intercellular to ambient CO_2_ concentration
*g*_s_	Stomatal conductance (mol m^−2^ s^−1^)
*A*_net_	Net photosynthetic rate (µmol m^−2^ s^−1^)
*E*	Transpiration rate (mmol m^−2^ s^−1^)
TE_intrinsic_	Intrinsic transpiration efficiency (*A*_net_/*g*_s_)
TE_instantaneous_	Instantaneous transpiration efficiency (*A*_net_/*E*)
TE_plant_	Plant level transpiration efficiency (total aboveground biomass/water transpired)
TE_w_	δ^13^C_BL_-derived transpiration efficiency
*e*_a_/*e*_i_	Molar ratio of ambient to intercellular vapor
ε^+^	Equilibrium fractionation
ε_k_	Kinetic fractionation
ϕ_w_	Ratio of night-time and non-stomatal water loss to daytime transpiration
ϕ_r_	Ratio of CO_2_ respiration occurring at night and in non- photosynthetic tissue during the day to assimilation rate
*A*	Fractionation during diffusion of CO_2_ in air through stomata (4.4‰)
*b*_*3*_	Fractionation by Rubisco (30‰)
*b*_*4*_	Fractionation of PEP carboxylation and isotopic equilibrium during dissolution and hydration of CO_2_ (–5.2‰ at a leaf temperature of 30 °C)
*S*	Fractionation during the CO_2_ leakage from the bundle sheath cells (1.8‰)
ϕ	Leakiness of CO_2_ from the bundle sheath

Variation in δ^13^C_BL_ in plants grown under the same climatic conditions is primarily determined by leaf photosynthetic CO_2_ isotope discrimination:

$$\Delta^{13}C_{\text{BL}}=\;\frac{\delta^{13}C_{\text{ambient}}-\delta^{13}C_{\text{BL}}}{1+\frac{\delta^{13}C_{\text{BL}}}{1000}}$$ (1)

where δ values are in ‰ notation and δ^13^C_ambient_ is the signature of available atmospheric CO_2_. In C_4_ plants, Δ^13^C is influenced by fractionations associated with diffusion of CO_2_, carboxylation reactions, and the ratio of bundle sheath CO_2_ leak rate to PEP carboxylase rate (leakiness, ϕ), and it is proportional to the partial pressure of intercellular to ambient CO_2_ (*C*_i_/*C*_a_). *C*_i_/*C*_a_ is a measure of the supply of CO_2_ to photosynthesis, and as *C*_i_/*C*_a_ increases, discrimination decreases (for the simplified model; von Caemmerer *et al.*, 2014):

$$\Delta^{13}C_{\text{BL}}=a+\left(b_{4}+\phi \left(b_{3}-s\right)-a\right)\frac{C_{i}}{C_{a}}$$ (2)

where *a* is the fractionation during diffusion of CO_2_ in air through stomata (4.4‰), *b*_4_ is the combined fractionation of PEP carboxylation and the preceding isotopic equilibrium during dissolution and hydration of CO_2_ (–5.2‰ at a leaf temperature of 30 °C) as described in Henderson *et al.* (1992), *b*_3_ is the fractionation by Rubisco (30‰), *s* is the fractionation during the leakage of CO_2_ out of the bundle sheath cells (1.8‰), and ϕ is the leakiness of CO_2_ from the bundle sheath (Henderson *et al.*, 1992, 1998).

The CO_2_ concentrating mechanism in C_4_ plants minimizes Rubisco fractionation, so the relationship between Δ^13^C and *C*_i_/*C*_a_ in C_4_ plants is dampened compared with C_3_ plants and is less variable across growth conditions and genotypes (Henderson *et al.*, 1998). Leakiness (ϕ) determines the slope of the relationship between Δ^13^C and *C*_i_/*C*_a_, controlling the directionality of this relationship from positive to negative. However, ϕ has been shown to be relatively constant in many C_4_ species, varying little across light intensities, temperatures, and CO_2_ partial pressures (Ubierna *et al.*, 2011; Sun *et al.*, 2012; Kromdijk *et al.*, 2014; Sage, 2014). Therefore, if ϕ is relatively robust and constant across different growth conditions, then changes in Δ^13^C are primarily driven by variation in *C*_i_/*C*_a_, which is influenced by both the net rates of CO_2_ fixation (*A*_net_) and stomatal conductance (*g*_s_). For example, increasing *A*_net_ can draw down *C*_i_ relative to *C*_a_ and a reduction in *g*_s_ can decrease the supply of atmospheric CO_2_ to the intercellular air space for photosynthetic assimilation. Since TE is also related to *A*_net_ and *g*_s_, this means that TE and Δ^13^C are linked through their relationship with *C*_i_/*C*_a_, which makes δ^13^C_BL_ a potential proxy for TE (Farquhar *et al.*, 1989; Henderson *et al.*, 1998).

Alternatively, oxygen isotopic enrichment above source water in leaf tissue (Δ^18^O_BL_) comes partly from oxygen isotopic enrichment in leaf lamina water, a component of leaf water enrichment (Δ^18^O_LW_), and where organic compounds are synthesized within the leaf. At these sites of carbonyl oxygen isotope exchange, the leaf water oxygen isotope signal is passed on to photosynthetic intermediates and consequently passed on to bulk leaf tissue (Barbour *et al.*, 2000*a*, *b*; Gan *et al.*, 2003; Barbour and Farquhar, 2004; Barbour, 2007). The model of Craig and Gordon (1965), which describes the evaporative isotopic enrichment of water from the surface of a water body, was modified to explain how oxygen isotopes in leaf water are enriched:

$$\Delta^{18}O_{e}=\varepsilon^{+}+\varepsilon_{k}+\frac{e_{a}}{e_{i}}\left(\Delta^{18}O_{v}-\varepsilon_{k}\right)$$ (3)

where isotopic enrichment of water at the evaporation sites (Δ^18^O_e_) is influenced by the isotopic enrichment above source water of ambient vapor (Δ^18^O_v_), temperature-dependent gradient in the molar ratio of ambient to intercellular water vapor (*e*_a_/*e*_i_), temperature-dependent equilibrium fractionation (ε^+^), and kinetic fractionation (ε_k_), which is a function of stomatal and boundary layer conductances (Eq. 3 and more in-depth explanation in Appendix).

The Craig–Gordon model describes Δ^18^O_e_, but tends to overestimate Δ^18^O_LW_. To account for this overestimation, the Craig–Gordon model was modified to account for unenriched leaf xylem water and the mixing behavior between xylem and lamina water pools (Péclet and two-pool models; Farquhar *et al.*, 1998; Roden and Ehleringer, 1999). The Péclet model suggests that δ^18^O_LW_ reflects the relative isotopic contributions of advection of source water in the xylem and back diffusion of water from the sites of evaporation. The proportional mixing of source water and water from the evaporation sites is primarily determined by the transpiration rate (*E*) and the mean effective path length (*L*) through which water passes from the xylem to the stomates (Farquhar and Lloyd, 1993; Barbour and Farquhar, 2004). Therefore, if *L* remains constant, Δ^18^O_LW_ decreases as *E* increases by decreasing the influence of Δ^18^O_e_ on Δ^18^O_LW_. Because Δ^18^O_BL_ partly reflects Δ^18^O_LW_, in the Péclet model where Δ^18^O_LW_ is related to *E*, Δ^18^O_BL_ can potentially provide an integrated proxy of *E* over the life of the leaf (Barbour *et al.*, 2000b; Barbour, 2007), and when coupled with biomass measurements can be a proxy for TE (Barbour *et al.*, 2000*a*). However, support for the Péclet effect has not been found in many instances (Song *et al.*, 2013; Roden *et al.*, 2015; Song *et al.*, 2015; Cernusak *et al.*, 2016; Holloway‐Phillips *et al.*, 2016). Therefore, it is uncertain if Δ^18^O_BL_ can be used as a proxy for *E* in C_4_ grasses.

In this study we tested the relationship between δ^13^C_BL_ and Δ^18^O_BL_ with TE and *E*, respectively, in the model C_4_ grasses *Setaria viridis* (L.) P. Beauv. and *S. italica* (L.) P. Beauv. These species are part of the C_4_ panicoid grass clade and are closely related to important food and biofuel crops, such as sugar cane, maize, miscanthus, and sorghum (Brutnell *et al.*, 2010; Li and Brutnell, 2011). *S. viridis* is a unique model organism for this clade because it has a short lifespan, a sequenced genome and a single nucleotide polymorphism (SNP) map for quantitative trait locus analysis (Doust *et al.*, 2009; Bennetzen *et al.*, 2012). Additionally, *S. viridis* and *S. italica* are drought-resistant species, growing in areas that cannot support sorghum, sugar cane, or maize production (Li and Wu, 1996). In the current study, both species were grown under well-watered and water-limited conditions to determine the effect that water limitations had on δ^13^C_BL_ and Δ^18^O_BL_ to evaluate their use as proxies for *g*_s_, *E*, and TE.

## Material and methods

### Growth and greenhouse conditions

#### *Experiment with* Setaria viridis

*Setaria viridis* (L.) P. Beauv. (accession A-10) was grown in the greenhouses at Washington State University, Pullman, WA, USA between June and July of 2013. Day and night temperatures were 26–30 and 21–25 °C, respectively. Daytime and night-time relative humidity were 30–57% and 59–89%, respectively. Plants received 500–1500 µmol m^−2^ s^−1^ photosynthetic photon flux density (PPFD) over 14 h. Pot distribution in the greenhouse was randomized every day to minimize the influence of lighting heterogeneity. Fifteen *S. viridis* seedlings per treatment with one seedling per pot were transplanted into 11.3-liter pots using a commercial potting soil mix (Sunshine LC 1). Plants received 20-20-20 NPK with micronutrients (JR Peters Inc., Allentown, PA 18106) twice weekly at 2.8 g l^−1^ water.

After the initial watering at transplanting, pots in the well-watered and water-limited treatments were maintained at a gravimetric water content (GWC) of ~4 and 1 g water g soil^−1^, respectively. In GWC calculations, fresh biomass would be considered soil water weight, causing GWC to be overestimated, so throughout the experiment daily transpiration was used as a proxy for changes in fresh weight over time to accurately calculate the soil water volume and estimates of GWC. All pots and lateral holes were covered with plastic sheeting to minimize soil evaporation. Daily transpiration was measured as the difference in pot weight between dawn and dusk minus soil evaporation (difference in a covered pot weight without a plant). All of the plants could not feasibly be measured and harvested in one day, so the plants were randomly divided into three collection groups of five plants from each treatment. Gas exchange measurements and harvesting of plant material of collections 1, 2, and 3 took place on 39, 43, and 44 d after germination. At all collection times, panicles had begun to emerge.

#### *Experiment with* Setaria italica

*Setaria italica* (L.) P. Beauv. (accession B-100; (Li and Brutnell, 2011) was grown in a controlled-environment growth cabinet (Enconair Ecological GC-16). Growth conditions were set at 16 h photoperiod including a 2 h ramp at dawn and dusk and maximum PPFD of 1000 µmol quanta m^−2^ s^−1^. Day and night temperatures were maintained at 28 ± 1 and 18 ± 1 °C, respectively and a mean relative humidity of 59 ± 6%. Pot location was randomized every day. A total of 33 *S. italica* seedlings (11 plants per treatment with one seedling per pot) were transplanted into 7.5-liter pots at 15 d after germination. The potting soil was the same as was used with *S. viridis*. Plants received 15-5-15 CalMag (JR Peters Inc., Allentown, PA, USA) twice weekly at a rate of 2.5 g l^−1^ water, Sprint 330 iron chelate (0.25 g l^−1^) weekly, and Scott-Peters Soluble Trace Element Mix, 10.0 mg l^−1^ biweekly (The Scotts Co., OH, USA).

After initial watering after transplanting, the GWC of the well-watered, moderately and severely water-limited treatments was maintained at 4.0, 0.9, and 0.5 g water g soil^−1^, respectively. Pots were covered with plastic similar to *S. viridis*. Six and five plants from each treatment were randomly selected to be harvested in the first and second collections, respectively. Leaf gas exchange measurements were made at six time points (31, 34, 40, 43, 53, and 54 d after germination). Plant material for stable isotope analysis was only collected at the final harvest, immediately following gas exchange measurements. At all collection times, panicles had begun to emerge in all plants.

### Gas exchange measurements

Measurements were made on the uppermost fully expanded leaf between 11:00 h and 15:00 h. Leaves were placed in a 2 × 3 chamber of an LI-6400XT open gas exchange system (Li-COR Biosciences, Inc., Lincoln, NE, USA). The leaf was allowed to acclimate at 1500 µmol m^−2^ s^−1^ PPFD, leaf temperature of 29 °C, flow rate of 300 µmol s^−1^, 21% O_2_, 35 Pa CO_2_ for *S. viridis*. The same conditions were used for *S. italica* except that light intensity was 900 µmol m^−2^ s^−1^ PPFD to reflect the light intensity of their growing conditions. Relative humidity (RH) in the LI-COR chamber was within 10% of the RH under growth conditions, and the implications of this difference in RH is explained in the next section.

### Sample collection for stable isotope analysis

To obtain sufficient leaf water to measure δ^18^O of leaf water (δ^18^O_LW_) from *S. viridis*, an aggregate of five to eight leaves (including the leaf used to measure gas exchange) was collected from each plant at the time of harvest. However, in *S. italica* the leaf used for gas exchange measurements was sufficient to analyse δ^18^O_LW_. The same leaf samples that were analysed for δ^18^O_LW_ were also analysed for leaf carbon isotopic composition (δ^13^C_leaf_) and oxygen isotopic composition of bulk leaf tissue (δ^18^O_BL_). All leaves collected for stable isotope analysis were the youngest, fully expanded leaves, which developed 15–20 d after soil water content reached the treatment set point. For both species, leaves were removed from the plant and photographed to measure leaf area using ImageJ software (Schneider *et al.*, 2012). Photographing each leaf only took approximately 20 s, and then the leaf was stored in sealed glass tubes awaiting water extraction.

In *S. italica* only, using the same leaf for gas exchange measurements and stable isotope analysis could have an effect on δ^18^O_LW_ because RH and the oxygen isotope ratio in the growth cabinet air (δ^18^O_v_) could differ between growth and gas exchange chambers (Loucos *et al.*, 2015). However, gas exchange measurements were conducted within the growth chamber, and the mean±SE proportion of the total leaf area continuously exposed to growth cabinet conditions during gas exchange measurements was 94.0 ± 0.6%, 91.0 ± 0.8%, and 89.5 ± 0.8% for well-watered, moderately water-limited, and severely water-limited treatments, respectively. Therefore, the δ^18^O of the growth cabinet air was the most appropriate measure of δ^18^O_v_. The difference in RH between the growth cabinet and the gas exchange chamber could contribute a 1.9–5.5‰ shift in δ^18^O_e_ for the leaf section in the gas exchange chamber, assuming, however unlikely, that 10–20 min was adequate time for the leaf section to acclimate. This would represent a shift in δ^18^O_e_ for the entire leaf of 0.24–0.43‰, and Δ^18^O_LW_ would shift by a fraction of this. Nonetheless, taking into account this potential shift did not significantly influence observed Δ^18^O_LW_ values across treatments. Differences between greenhouse and LI-COR chamber conditions for *S. viridis* were irrelevant because leaf water was extracted from an aggregate of five to eight leaves.

The δ^18^O of water vapor in the greenhouse and growth chambers was measured every 30 min during gas exchange measurements by collecting air in 5-liter Supel inert foil gas sampling bags (Supelco, Bellefonte, PA, USA). Bags were flushed several times with air before filling, and δ^18^O of water vapor was immediately measured on the cavity ringdown spectrometer (L1102-*i* water analyser, Picarro Inc, Santa Clara, CA, USA).

Following the gas exchange measurements, root crowns were cleaned of soil and stored at −20 °C in air-tight tubes. Root crowns are considered the bottom 1 cm of the culm or tiller, but no actual roots were collected. Additionally, two soil samples were collected at the top and bottom of each pot, and stored using the same method as with the root crowns. Leaves, soils, and root crowns were distilled using a cryogenic vacuum distillation method (Vendramini and Sternberg, 2007). Additionally, daily samples of irrigation water (IW) were collected to measure δ^18^O_IW_.

### Biomass measurements

After collection of plant tissue for stable isotope analysis, the entire aboveground biomass was collected and weighed for fresh weight. Samples were dried at 65 °C for 3 d before weighing dry biomass.

### Stable isotope analysis

The stable isotope composition of carbon and oxygen (δ^18^O and δ^13^C, respectively) were reported in δ notation in parts per thousand (‰),

$$\delta =\;\left(\frac{R_{\text{sample}}}{R_{\text{standard}}}-1\right)$$ (4)

where *R*_sample_ and *R*_standard_ are the molar ratios of heavy to light isotope (^18^O/^16^O and ^13^C/^12^C) of the sample and international standard, respectively. The international standard used for oxygen was Vienna Standard Mean Ocean Water (VSMOW) and for carbon was Vienna Pee Dee Belemnite (VPDB).

### Stable isotope analysis by isotope ratio mass spectrometer

Leaf tissue was analysed for oxygen isotopic analysis by converting to CO with a pyrolysis elemental analyser (TC/EA, Thermo Finnigan, Bremen, Germany) and analysed with a continuous flow isotope ratio mass spectrometer (Delta PlusXP, Thermo Finnigan; Brenna *et al.*, 1997; Gehre and Strauch, 2003). For carbon isotopic analysis, leaf tissue was converted to CO_2_ with an elemental analyser (ECS 4010, Costech Analytical, Valencia, CA, USA) and analysed with a continuous flow isotope ratio mass spectrometer (Delta PlusXP; Brenna *et al.*, 1997; Qi *et al.*, 2003). Isotopic standards, Standard Light Antarctica Precipitation (SLAP; Gonfiantini, 1978) and Puerto Rico water (Qi *et al.*, 2014), were analysed alongside samples to calculate δ^18^O based on the VSMOW scale. Lab standards, calibrated to international standards, were used to calculate δ^13^C relative to VPDB. Standard error for δ^13^C values for the *S. viridis* and *S. italica* experiments was 0.11 and 0.05‰, respectively. The standard error for δ^18^O was 0.2‰ for both experiments.

Leaf and root crown water were analysed for oxygen isotope composition by equilibrating 0.5 ml of water at room temperature with 0.3% CO_2_:He mixture for 48 h on a ThermoFinnigan GasBench II (Thermo Electron Corp., Bremen, Germany). CO_2_ was analysed with a continuous flow isotope ratio mass spectrometer (Delta PlusXP; Brenna *et al.*, 1997; Qi *et al.*, 2003).

### Stable isotope analysis by isotope ratio infrared spectroscopy

Soil and irrigation water were measured by isotope ratio infrared spectroscopy (model L1102-*i*, Picarro, Sunnyvale, CA, USA) connected to a vaporization chamber (V1102-*i*). The mean δ^18^O was calculated of the last three of six consecutive analyses on each sample. Three laboratory standards, calibrated to the VSMOW scale, were interspersed among the samples and were used to correct the sample δ^18^O values to the VSMOW scale. Water vapor was analysed for at least 15 min, but the mean of the last 5 min was used for δ^18^O and corrected to the VSMOW scale.

### Calculations of transpiration efficiency

TE_instantaneous_ (*A*_net_/*E*) and TE_intrinsic_ (*A*_net_/*g*_s_) are derived from gas exchange measurements, but they are independent of both TE_plant_ and TE_w_, which are independent of each other. TE_plant_ was derived from whole-plant measures of biomass and transpiration. TE_w_ was calculated using *C*_i_/*C*_a_ derived from δ^13^C_BL_, and the calculations are independent of the gas exchange measurements (described below).

Discrimination (Δ^13^C_BL_) and *C*_i_/*C*_a_ are related in Eq. 2 and were used to calculate the integrated *C*_i_/*C*_a_ over the life of the leaf (Henderson *et al.*, 1992, 1998). A constant leakiness (ϕ) of 0.21 was assumed for all plants. The δ^13^C of ambient CO_2_ in the greenhouse and growth chamber that was used to calculate Δ^13^C_BL_ was −10.7 ± 0.8‰, which was collected various times over a period of several months that included the time that the experiment was conducted. Air samples were collected in 5-liter Tedlar gas sampling bags using the same collection procedure used to collect air vapor. The gas was analysed by introducing air directly into either the isotope ratio mass spectrometer or the tunable diode laser absorption spectroscope (model TGA 200A, Campbell Scientific, Inc., Logan, UT, USA; Ubierna *et al.*, 2013). Transpiration efficiency (TE_w_), defined as the ratio of dry matter produced per unit of water transpired, was calculated from the δ^13^C_BL_ as described in Henderson *et al.* (1998) as:

$${\text{TE}}_{w}=\left(1-\frac{C_{i}}{C_{a}}\right)\times \frac{C_{a}}{1.6v}\times \frac{\left(1-\phi_{r}\right)}{\left(1-\phi_{w}\right)}$$ (5)

where *v* is the leaf to air vapor pressure difference and ϕ_r_ was calculated as the measured ratio of respiration and photosynthetic rate (0.08 for all plants). This parameter was calculated from gas exchange measurements made during the experiment. The parameter ϕ_w_ was calculated as the measured ratio of whole-plant night to day transpiration (0.30 and 0.23 for water-limited and well-watered plants, respectively) measured in this experiment. Both night-time and daytime whole-plant transpiration were measured on *S. viridis*. Both ϕ_r_ and ϕ_w_ were measured for *S. viridis* and assumed to be the same for *S. italica*. *C*_i_/*C*_a_ was calculated from δ^13^C_BL_ using Eq. 2.

### Model calculations to determine validity of the Péclet model

To test the applicability of the Péclet model and its relationship to *E*, we used the method described by Holloway-Phillips *et al.* (2016) and Song *et al.* (2015) of determining the proportional deviation (*f*) of Δ^18^O_LW_ from Δ^18^O_e_ plotted against *E* where *f* is calculated as:

$$f=1-\;\frac{\Delta^{18}O_{\text{LW}}}{\Delta^{18}O_{e}}$$ (6)

Effective path length (*L*) was calculated using the equations described in Song *et al.* (2013).

### Statistical analysis

In both experiments statistical analyses were conducted in R v. 3.3.0 (R Core Team, 2013), using car (v. 2.0-26) and agricolae (version 1.2-2) packages for statistical tests. Model II regressions (standard major axis regression) were calculated, using the lmodel2 (v. 1.7-2) package, because neither variable was controlled, both varied naturally with their own associated error, and the physical units of both variables were not the same. Homogeneity was tested based on plotting predicted fit *vs* residuals. Using the extRemes package (v. 2.0-8), normality was tested by plotting residuals on quantiles–quantiles plots. In all cases, where normality was questionable, transforming the data did not change the statistical results, so the data were not transformed. One- and two-way analysis of variance (ANOVA) was used to determine differences across treatments in the experiment with *S. italica* and between treatments and collection periods for *S. viridis*. Two-sample Student’s *t*-tests were performed to determine the difference between δ^18^O_SW_ and δ^18^O_RC_ values. One-sample *t*-tests were performed to determine if the difference of both δ^18^O_SW_ and δ^18^O_RC_ with δ^18^O_IW_ was significantly different from 0. Repeated measures ANOVAs were conducted on the following parameters: total plant water use and GWC for *S. viridis* and on daily water use, GWC, *A*_net_, *E*, and *g*_s_ for *S. italica*.

## Results

### Plant growth and treatment effect

**Fig. 1.**
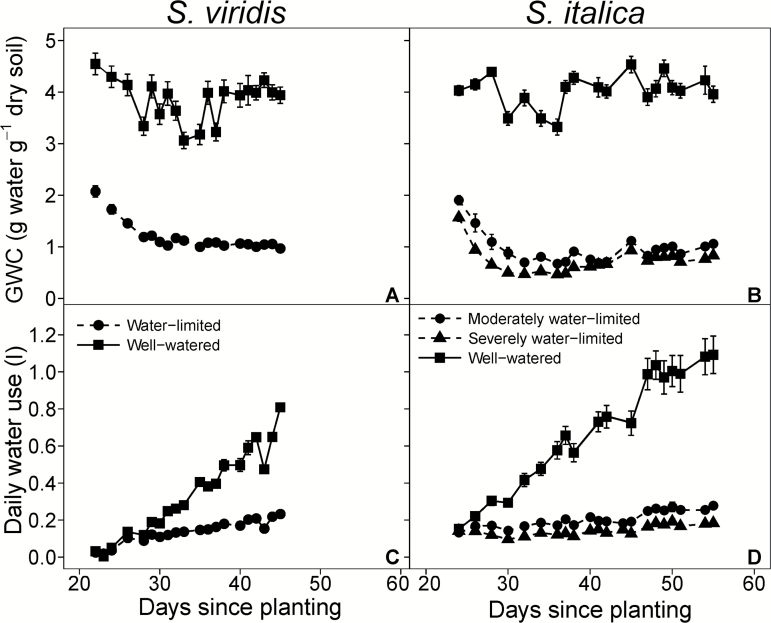
Gravimetric water content (GWC; A, B) and daily water use (C, D) over the course of the experiment. Circles, triangles, and squares represent well-watered, moderately water-limited and severely water-limited plants, respectively. Solid and dashed lines represent well-watered and water-limited treatments, respectively. Error bars represent standard error.

Maintaining water-limited plants at a GWC 71% lower than the well-watered plants significantly reduced total water use by 59% (Table 2 and Fig. 1). Additionally, at all collection times fresh and dry aboveground biomass were lower in the water-limited treatment relative to the well-watered treatment (Table 3). The results for *S. italica* were similar to *S. viridis* in that the GWC was reduced by 72% and 82%, and total water use by 70% and 80% in the moderately and severely water-limited treatments, respectively (Table 2; Fig. 1). In *S. italica*, fresh and dry aboveground biomass were reduced in the moderately water-limited, 71% and 68%, respectively, and in the severely water-limited treatment, 79% and 74%, respectively (Table 4; Fig. 1). Additionally, leaf length in the water-limited treatments was shorter compared with the well-watered leaves by 18% in *S. viridis* and in *S. italica* by 32% and 57% in the moderately and severely water-limited treatments, respectively (Tables 3 and 4). The number of tillers per plant in the well-watered treatment was 44% greater than the water-limited treatment in *S. viridis* (Table 3); however, the number of tillers per *S. italica* plant did not differ across treatments (Table 4).

**Table 2.** Statistical summary for repeated-measures ANOVA of variables measured throughout the experiments Levels of significance were calculated from two-factor repeated measures ANOVA described in ‘Materials and methods’, *P<0.05, **P<0.01, and ***P<0.001, ns not significant (*P*>0.05).

Level	Variables	Species	Between effects		Within effects			
			Treatment		Date		Date × Treatment	
			*F*_ndf,ddf_	*P*	*F*_ndf,ddf_	*P*	*F*_ndf,ddf_	*P*
Plant	Water use (l H_2_O plant^−1^)	*S. viridis*	180.8_1,28_	***	325.8_16,448_	***	16.73_16,448_	***
		*S. italica*	187.0_2,29_	***	119.3_18,522_	***	69.01_36,522_	***
	GWC (g water g dry soil^−1^)	*S. viridis*	205.9_1,28_	***	97.6_16,448_	***	30.5_1,448_	ns
		*S. italica*	312.3_2,29_	***	45.1_2,29_	***	5.235_36,522_	***
Leaf	*C*_i_/*C*_a_	*S. italica*	33.67_2,16_	***	0.443_4,64_	ns	1.464_8,64_	ns
	*E* (mmol^−1^ H_2_O m^−2^ s^−1^)	*S. italica*	73.5_2,18_	***	4.07_5,98_	ns	1.008_10,98_	ns
	*g*_s_ (mmol^−1^ H_2_O m^−2^ s^−1^)	*S. italica*	60.8_2,18_	***	1.869_5,98_	ns	1.644_10,98_	ns
	*A*_net_ (µmol^−1^ CO_2_ m^−2^ s^−1^)	*S. italica*	9.834_2,18_	**	2.15_5,98_	ns	0.988_10,98_	ns

**Table 3.** *Parameters of plant water relations and isotopic composition of* S. viridis Each variable was analysed with a separate two-factor ANOVA. The Δ^18^O_LW_ and Δ^18^O_BL_ values were calculated using each of the possible water sources (source water in parentheses). *P*-values for the ANOVAs are located on the right side (*P<0.05, **P<0.01, and ***P<0.001, ns not significant). Columns 1, 2 and ‘I’ are the factors ‘differential irrigation’ (1), ‘collection time’ (2), and the differential irrigation × collection time interaction (I). Means±SE within a row, followed by the same superscripted letters, are not significantly different. Other factors are given in Supplementary Tables S1 and S2 at *JXB* online.

Parameters	Collection 1		Collection 2		Collection 3		ANOVA		
	Well-watered	Water-limited	Well-watered	Water-limited	Well-watered	Water-limited	1	2	I
Total water use (l)	5.33 ± 0.38	2.13 ± 0.06	6.97 ± 0.29	2.86 ± 0.06	7.94 ± 0.56	3.24 ± 0.2	***	***	ns
Fresh aboveground biomass (g)	192.3 ± 10.7	74.7 ± 2.1	229.4 ± 5.1	87.8 ± 1.3	234.5 ± 13.8	77.0 ± 1.6	***	**	ns
Dry aboveground biomass (g)	31.8 ± 2.1	16.1 ± 0.4	41.6 ± 2.2	21.0 ± 0.3	45.4 ± 3.3	22.6 ± 0.6	***	***	ns
Number of tillers	125 ± 6	74 ± 3	142 ± 4	78 ± 4	145 ± 17	77 ± 1	***	ns	ns
Δ^18^O_LW_ (root crown)	21.0 ± 0.3^c^	21.8 ± 0.7^bc^	19.5 ± 0.4^c^	21.7 ± 0.6^bc^	23.8 ± 0.5^b^	29.4 ± 0.7^a^	***	***	**
Δ^18^O_LW_ (soil)	20.4 ± 0.3	22.4 ± 0.6	19.4 ± 0.4	21.7 ± 0.6	24.2 ± 0.6	28.8 ± 0.5	***	***	ns
Δ^18^O_LW_ (irrigation)	21.4 ± 0.3^cd^	24.0 ± 0.8^b^	20.1 ± 0.5^d^	23.3 ± 0.5^bc^	25.0 ± 0.5^b^	31.7 ± 0.6^a^	***	***	**
Δ^18^O_BL_ (root crown)	43.5 ± 1.1	45.2 ± 0.8	43.6 ± 0.5	44.2 ± 0.6	44.7 ± 0.8	45.4 ± 0.7	ns	ns	ns
Δ^18^O_BL_ (soil)	43.2 ± 0.9	45.8 ± 0.8	43.6 ± 0.6	44.2 ± 0.6	45.1 ± 0.8	44.9 ± 0.5	ns	ns	ns
Δ^18^O_BL_ (irrigation)	44.2 ± 0.9	47.3 ± 0.7	44.2 ± 0.7	45.7 ± 0.6	45.7 ± 0.8	47.7 ± 0.6	**	ns	ns
δ^13^C_BL_	−13.4 ± 0.1	−14.4 ± 0.1	−13.4 ± 0.1	−14.5 ± 0.1	−13.5 ± 0.04	−14.6 ± 0.1	***	ns	ns
δ^18^O_RC_	−16.5 ± 0.2	−14.9 ± 0.2	−16.5 ± 0.2	−15.5 ± 0.2	−15.9 ± 0.1	−14.7 ± 0.2	***	**	ns
δ^18^O_SW_	−16.0 ± 0.1^b^	−15.5 ± 0.2^b^	−16.4 ± 0.1^b^	−16.3 ± 0.3^b^	−16.3 ± 0.2^b^	−14.1 ± 0.2^a^	***	*	**
*C*_i_/*C*_a_	0.41 ± 0.03	0.26 ± 0.06	0.37 ± 0.02	0.18 ± 0.02	0.36 ± 0.02	0.13 ± 0.02	***	*	ns
*g*_s_ (mol H_2_O m^−2^ s^−1^)	0.26 ± 0.01^a^	0.22 ± 0.02^a^	0.26 ± 0.01^a^	0.15 ± 0.02^b^	0.26 ± 0.02^a^	0.06 ± 0.01^c^	***	***	***
*E* (mmol H_2_O m^−2^ s^−1^)	5.03 ± 0.23^ab^	4.57 ± 0.19^b^	4.88 ± 0.14^ab^	3.27 ± 0.36^c^	5.6 ± 0.16^a^	1.87 ± 0.19^d^	***	***	***
*A*_net_ (µmol CO_2_ m^−2^s^−1^)	28.3 ± 0.8^ab^	32.3 ± 1.2^a^	31.0 ± 0.6^ab^	25.4 ± 2.2^b^	31.2 ± 0.6^a^	12.1 ± 1.5^c^	***	***	***
Water/leaf area (l H_2_O m dry leaf^−2^)	0.14 ± 0.01	0.13 ± 0.01	0.12 ± 0.003	0.11 ± 0.002	0.11 ± 0.001	0.10 ± 0.01	ns	***	ns
TE_instantaneous_ (*A*_net_/*E*)	5.68 ± 0.31	7.13 ± 0.47	6.36 ± 0.12	7.86 ± 0.24	5.59 ± 0.11	6.44 ± 0.31	***	**	ns
TE_intrinsic_ (*A*_net_/*g*_s_)	109.0 ± 5.6	151.6 ± 12.8	121.1 ± 3.8	170.1 ± 5.3	123.5 ± 5.4	193.4 ± 5.1	***	**	ns
TE_plant_ (g biomass l H_2_O^−1^)	5.98 ± 0.09	7.57 ± 0.10	5.96 ± 0.09	7.35 ± 0.09	5.69 ± 0.08	7.06 ± 0.16	***	ns	ns
TE_w_ (derived from δ^13^C_BL_)	5.47 ± 0.22	7.25 ± 0.13	5.49 ± 0.10	7.43 ± 0.22	5.64 ± 0.07	7.61 ± 0.09	***	ns	ns
Effective leaf length (mm)	13.58 ± 1.9^a^	10.84 ± 3.9^ab^	16.88 ± 2.1^a^	11.92 ± 2.2^a^	9.16 ± 4.1^ab^	1.76 ± 1^b^	*	**	ns
Leaf length (cm)	19.2 ± 0.2	17.5 ± 0.1	19.9 ± 0.8	16.7 ± 0.4	19.6 ± 0.7	15.7 ± 0.2	***	ns	ns

**Table 4.** *Plant water relations, growth, and stable isotopes of* S. italica *grown under well-watered, moderately and severely water-limited treatments and harvested during two collection periods* One-way ANOVAs were conducted on each variable as described in ‘Materials and methods’. The source water used to calculate Δ^18^O_LW_ and Δ^18^O_BL_ was root crown water. *P*-values are reported in the right column (***P<0.001, **P<0.01, *P<0.05, ns *P*>0.05). Means±SE were calculated for each variable, and within the same row, means followed by the same letter were not significantly different. Other factors also were analysed and are given in Supplementary Tables S3 and S4.

Variables	Well-watered	Water limitation		ANOVA
		Moderate	Severe	*P*
Total water use (l)	12.65 ± 0.88^a^	3.77 ± 0.07^b^	2.57 ± 0.06^c^	***
Fresh aboveground biomass (g)	486.1 ± 36.4^a^	140.7 ± 6.3^b^	100.4 ± 3.3^c^	***
Dry aboveground biomass (g)	104.7 ± 8.0^a^	33.4 ± 1.3^b^	24.9 ± 1.1^c^	***
Number of tillers	9.2 ± 1.6^a^	6.6 ± 1.0^a^	7.9 ± 0.9^a^	ns
Δ^18^O_LW_	21.5 ± 0.8^a^	20.8 ± 1.0^a^	20.6 ± 1.3^a^	ns
Δ^18^O_BL_	42.1 ± 0.5^a^	40.8 ± 0.4^ab^	40.1 ± 0.5^b^	*
δ^13^C_BL_	−12.96 ± 0.06^a^	−13.88 ± 0.10^b^	−13.81 ± 0.11^b^	***
δ^18^O_SW_	−16.2 ± 0.1^c^	−14.2 ± 0.2^b^	−13.0 ± 0.1^a^	***
δ^18^O_RC_	−15.9 ± 0.1^c^	−13.9 ± 0.2^b^	−12.7 ± 0.3^a^	***
*C*_i_/*C*_a_	0.44 ± 0.01^a^	0.30 ± 0.01^b^	0.27 ± 0.01^b^	***
*g*_s_ (mmol^−1^ H_2_O m^−2^ s^−1^)	0.173 ± 0.019^a^	0.119 ± 0.019^b^	0.110 ± 0.009^b^	***
*E* (mmol^−1^ H_2_O m^−2^ s^−1^)	2.13 ± 0.16^a^	1.59 ± 0.19^b^	1.48 ± 0.12^b^	***
*A*_net_ (µmol^−1^ CO_2_ m^−2^ s^−1^)	20.3 ± 1.4^a^	18.1 ± 2.1^b^	17.4 ± 0.8^b^	***
Water per leaf area (l H_2_O m^−2^ dry leaf)	0.19 ± 0.008^a^	0.17 ± 0.013^ab^	0.15 ± 0.009^b^	*
TE_instantaneous_ (*A*_net_/*E*)	9.6 ± 0.2^b^	11.4 ± 0.3^a^	11.8 ± 0.2^a^	***
TE_intrinsic_ (*A*_net_/*g*_s_)	118.4 ± 3.39^b^	153.2 ± 3.25^a^	158.1 ± 3.08^a^	***
TE_plant_ (g biomass l H_2_O transpired^−1^)	8.25 ± 0.22^b^	8.89 ± 0.34^ab^	9.69 ± 0.36^a^	*
TE_w_ (mmol C mol H_2_O^−1^; derived from δ^13^C_BL_)	4.94 ± 0.09^b^	6.49 ± 0.19^a^	6.37 ± 0.17^a^	***
Leaf length (cm)	43.9 ± 1.9^a^	33.2 ± 1.4^b^	28.0 ± 1.0^c^	***

For both species, stomatal conductance (*g*_s_), rates of transpiration (*E*), and the net rate of CO_2_ assimilation (*A*_net_) were generally higher in the well-watered treatment compared with the water-limited treatment. Gas exchange measurements of *S. viridis* were made during the three biomass collections, and *E* was 33% and 67% greater in the well-watered treatment in collections 2 and 3, respectively, but did not differ between treatments in collection 1 (Table 3). Additionally, *g*_s_ was 41% and 76% greater in the well-watered treatment during collection 2 and 3, respectively, but did not differ between treatments in collection 1. However, *A*_net_ in *S. viridis* was different between treatments only in collection 3 when *A*_net_ of water-limited plants was 61% lower than that of the well-watered treatment (Table 3). In *S. italica*, *E*, *g*_s_, and *A*_net_ were not different between the severely and moderately water-limited treatments, but both treatments were on average 27%, 32%, and 16% lower, respectively, than the well-watered treatment (Table 4).

### Leaf carbon isotopic composition

**Fig. 2.**
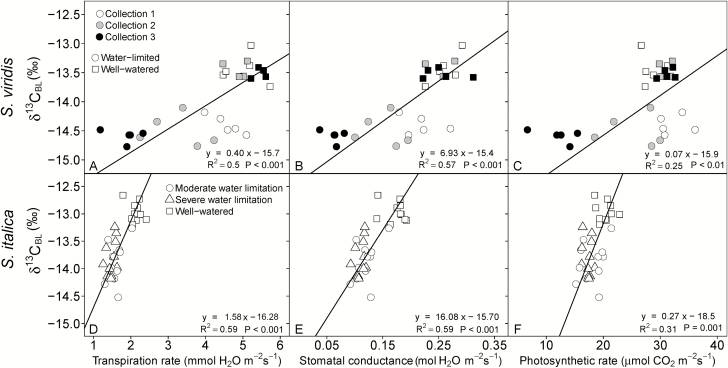
Linear relationship between δ^13^C_BL_ and transpiration rate (A, D), stomatal conductance (B, E), and photosynthetic rate (C, F) as measured at time of plant harvest. In the top panel, circles represent the water-limited treatment and squares represent well-watered plants, and open, gray-filled and black-filled symbols represent collection 1, 2, and 3, respectively. In the bottom panel, squares represent the well-watered treatment, circles represent the moderately water-limited treatment, and triangles represent the severely water-limited treatment. Lines represent Model II regression.

**Fig. 3.**
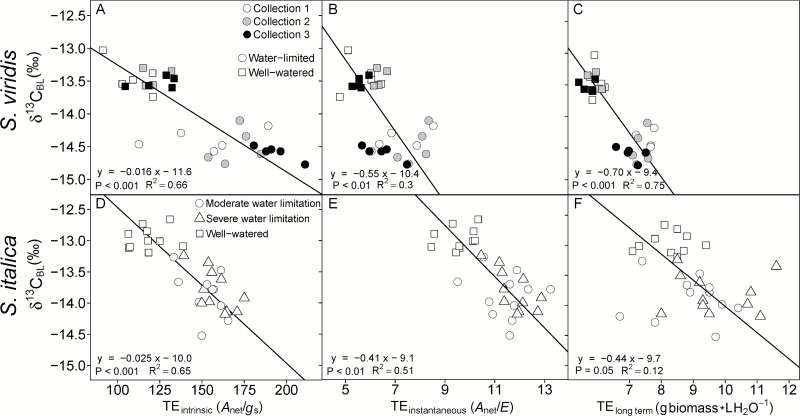
Intrinsic TE (A, D; *A*_net_/*g*_s_), instantaneous TE (B, E; *A*_net_/*E*) and long term TE (C, F; g aboveground biomass l H_2_O transpired^−1^) regressed on bulk leaf carbon isotopic composition (δ^13^C_BL_). Lines represent Model II regression. (A–C) are *S. viridis* and (D–F) are *S. italica*.

The response in leaf carbon isotopic signature (δ^13^C) to water limitations was similar for both species. For example, δ^13^C values in *S. viridis* were consistently lower in the water-limited treatment for all collections by 1.1‰ (Table 3) and in *S. italica* the δ^13^C values were 0.9‰ lower in the two water-limited treatments compared with the well-watered treatments (Table 4). The δ^13^C values of both species were positively correlated with *A*_net_, *E*, and *g*_s_, leaf water content, Δ^18^O_LW_, Δ^18^O_BL_, and all measurements of TE and plant growth and had stronger correlations with these parameters than either Δ^18^O_LW_ or Δ^18^O_BL_ (Table 5 and Figs 2 and 3).

**Table 5.** *Correlations between measured variables of both well-watered and water-limited plants and leaf water enrichment (Δ* ^*18*^ *O*_*LW*_ *), bulk leaf enrichment (Δ* ^*18*^ *O*_*BL*_ *), and δ* ^*13*^ *C*_*BL*_ *for* S. viridis *and δ*^*13*^*C*_*BL*_*for* S. italica The source water used to calculate Δ^18^O_LW_ and Δ^18^O_BL_ is listed in the header of each column. Significant correlations (*r*) are in bold, and the level of significance is given. Levels of significance are *P<0.05, **P<0.01, ***P<0.001. Other factors also were analysed and are given in Supplementary Tables S5 and S6. Correlations between Δ^18^O_LW_ and Δ^18^O_BL_ were only calculated where the same source water was used to calculate both

Parameter	*S. viridis*							*S. italica*
	Δ^18^O_LW_			Δ^18^O_BL_			δ^13^C_BL_	δ^13^C_BL_
	Irrigation water	Soil	Root crown	Irrigation water	Soil	Root crown		
*g*_s_	−0.75***	−0.57**	—0.71***	−0.50**	—0.26	−0.29	0.65***	0.77***
*E*	−0.66***	−0.46*	—0.61***	−0.41*	—0.22	−0.26	0.59***	0.77***
*A*_net_	−0.77***	—0.73***	—0.76***	−0.33	—0.06	−0.17	0.56**	0.56**
Fresh aboveground biomass	−0.47*	−0.44*	−0.36	−0.45*	—0.22	−0.19	0.87***	0.77***
Dry aboveground biomass	−0.26	−0.21	−0.15	−0.32	—0.13	−0.09	0.74***	0.76***
Total water transpired	−0.28	−0.23	−0.17	−0.33	—0.14	−0.10	0.85***	0.77***
Leaf water per area (l m^−2^)	−0.57**	−0.66**	—0.59**	−0.33	—0.32	−0.33	0.33	−0.02
TE_plant_	0.30	0.25	0.17	0.42*	0.29	0.18	—0.87***	**—** 0.39*
TE_instantaneous_	−0.05	−0.04	−0.16	0.26	0.36	0.16	—0.55**	—0.72***
TE_intrinsic_	0.64***	0.64***	0.56**	0.56**	0.35	0.35	—0.81***	—0.81***
δ^13^C_BL_	−0.60***	−0.54**	—0.50**	−0.59***	—0.36	−0.35		
Δ^18^O_BL_	0.63***	0.31	0.45*					

Using the simplified equation (Eq. 2) of Δ^13^C *versus C*_i_/*C*_a_, where Δ^13^C was calculated from δ^13^C_BL_ and *C*_i_/*C*_a_ was measured, the mean leakiness of 0.21 ± 0.02 and 0.19 ± 0.01 was calculated for *S. viridis* in the well-watered and water-limited treatments, respectively. In *S. italica* leakiness was 0.17 ± 0.01 for well-watered and severely water-limited treatments and 0.20 ± 0.01 in the moderately water-limited. Overall, leakiness did not significantly differ between species or across treatments (0.17–0.21). The difference in leakiness would account for 0.22 ± 0.03‰ and 0.24 ± 0.01‰ of the observed difference in Δ^13^C_BL_ between treatments over the observed range of *C*_i_/*C*_a_ in *S. italica* and *S. viridis*, respectively.

### **Transpiration efficiency (TE) and leaf δ** ^**13**^ **C-derived transpiration efficiency (TE**_**w**_)

Four different methods were used to calculate transpiration efficiency: (i) long term TE (TE_plant_; grams of aboveground dry biomass per liter water transpired); (ii) instantaneous TE (TE_instantaneous_; *A*_net_/*E*); (iii) intrinsic TE (TE_intrinsic_; *A*_net_/*g*_s_); and (iv) δ^13^C_BL_-derived TE (TE_w_; mmoles carbon fixed per mole H_2_O transpired). For *S. viridis* the water-limited plants had higher TE regardless of how it was estimated, except in collection 3 where there was no difference in TE_instantaneous_ between treatments (Table 3). In *S. italica*, all four estimates of TE were higher in both water-limited treatments than the well-watered treatment, but the difference was not significant between the moderately and severely water-limited treatments. In both species, the TE_intrinsic_ had the largest differences between treatments (45% and 32% greater in *S. viridis* and *S. italica*, respectively; Tables 3 and 4). Additionally, TE_intrinsic_ had the strongest relationship with Δ^18^O_LW_, Δ^18^O_BL_, and δ^13^C (Table 5).

### Leaf oxygen isotopic composition

The gas exchange measurements and leaf samples were collected between 11:00 and 15:00 h. During this time the vapor oxygen isotope ratios (δ^18^O_v_) in both the greenhouse for *S. viridis* and in the growth chambers for *S. italica* were relatively stable. For *S. viridis*, δ^18^O_v_ values (mean±SE) were −17.4 ± 0.2, −21.9 ± 0.4, and −24.2 ± 0.3 for the three collections, respectively. For *S. italica*, δ^18^O_v_ values were −25.8 ± 0.1 and −20.1 ± 0.8 for the two collections, respectively.

Plants were top irrigated in covered pots with irrigation water (−17.0 ± 0.1‰ and −16.9 ± 0.1‰ for *S. viridis* and *S. italica*, respectively). The mean δ^18^O_SW_ values (average of δ^18^O of soil samples from the top and bottom of the pot) of the well-watered and water-limited treatments were 0.8 ± 0.1‰ and 1.9 ± 0.2‰ higher than irrigation water for *S. viridis* (*P*<0.0001), respectively. For *S. italica*, δ^18^O_SW_ values were 0.8 ± 0.2‰, 2.7 ± 0.2‰, and 3.9 ± 0.4‰ higher than irrigation water in the well-watered, moderately and severely water-limited treatments, respectively (*P*<0.0001). In *S. viridis*, the δ^18^O_RC_ values were 0.6 ± 0.1‰ and 2.0 ± 0.1‰ higher than δ^18^O_IR_ in the well-watered and water-limited plants (*P*=0.0002 and *P*<0.0001), respectively. In *S. italica*, δ^18^O values were 1.1 ± 0.1‰, 3.1 ± 0.2‰, and 4.3 ± 0.3‰ higher than the irrigation water in the well-watered, moderately and severely water-limited treatments (*P*<0.0001), respectively. The δ^18^O of root crown and soil water was not significantly different in either *S. viridis* or *S. italica* (*P*>0.05).

**Fig. 4.**
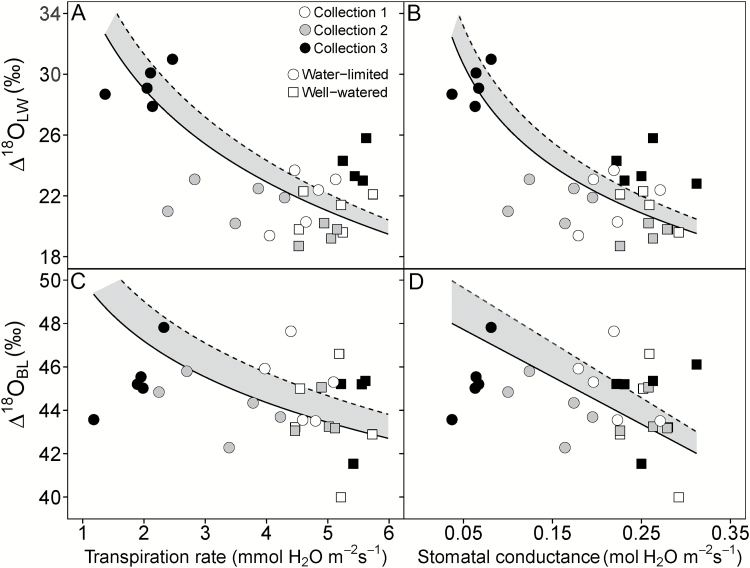
Relationship between Δ^18^O_LW_ (A, B) and Δ^18^O_BL_ (C, D) and transpiration rate (A, C) and stomatal conductance (B, D) in *S. viridis*. Gas exchange measurements were made at time of plant harvest. Circles represent the water-limited treatment and squares represent well-watered plants. Open, gray-filled and black-filled symbols represent collection 1, 2, and 3, respectively. The points represent Δ^18^O_LW_ and Δ^18^O_BL_ when they were calculated using δ^18^O_RC_ as source water (δ^18^O_LW_ or δ^18^O_BL_ minus δ^18^O_S_). The dashed regression line represents the regression when δ^18^O_I_ was used to calculate Δ^18^O_LW_ and Δ^18^O_BL_. The solid regression line represents the regression when δ^18^O_SW_ or δ^18^O_RC_ were used to calculate Δ^18^O_LW_ and Δ^18^O_BL_. The resulting regression line between Δ^18^O_LW_ or Δ^18^O_BL_ with transpiration rate (*E*) and stomatal conductance (*g*_s_) did not differ when δ^18^O_SW_ or δ^18^O_RC_ was used to calculate Δ^18^O_LW_ and Δ^18^O_BL_. Therefore, the shaded region represents the variation associated with which source water was used. Lines represent Model II regressions.

Leaf water enrichment (Δ^18^O_LW_) showed a significant treatment effect in *S. viridis* independent of which water was considered source water (irrigation, soil, or root crown water), but the treatment effect on Δ^18^O_LW_ was greatest with irrigation water. Likewise the strength of the correlation between parameters of gas exchange, water use, and growth depended on which source water was used to calculate Δ^18^O_LW_. However, independent of the source water, Δ^18^O_LW_ negatively correlated with *A*_net_, *E*, and *g*_*s*_ and positively correlated with TE_intrinsic_ and δ^13^C_BL_ (Table 5 and Fig. 4). In *S. italica*, Δ^18^O_LW_ did not have a significant treatment effect or correlate with growth, gas exchange, or TE variables, regardless of source water used (Table 4).

In *S. viridis*, a significant treatment effect in Δ^18^O_BL_ was only found and Δ^18^O_BL_ only correlated with *g*_s_, *E*, fresh aboveground biomass, TE_plant_, and TE_intrinsic_, Δ^18^O_LW_, and δ^13^C_BL_ when irrigation water was used as source water (Table 5 and Fig. 4). The only parameter that correlated significantly with Δ^18^O_BL_ when root crown water was considered the source water was Δ^18^O_LW_. For *S. italica*, the Δ^18^O_BL_ in the well-watered treatment was 2.0‰ greater than the severely water-limited treatment, but Δ^18^O_BL_ of the moderately water-limited treatment was not different from either treatment (Table 4). This difference resulted in significant positive correlations with *E* and *g*_s_ (0.60 and 0.59, respectively) when negative correlations were expected.

The Péclet model was tested by comparing the proportional deviation of Δ^18^O_LW_ from Δ^18^O_e_ (*f*; Eq. 6) with *E* (see Supplementary Fig. S1 at *JXB* online). This relationship was significant for *S. viridis* (*f*=0.086*E*–0.221, *R*^2^=0.36, *P*=0.0005) but not for *S. italica* (*P*=0.93) suggesting that in *S. viridis* Δ^18^O_LW_ deviated more from Δ^18^O_e_ as *E* increased. For *S. italica*, the Δ^18^O_LW_ was larger than Δ^18^O_e_ (negative *f* values), causing Δ^18^O_LW_ to be more enriched than would be expected based on the evaporative environment. For *S. viridis* the estimated effective leaf length (*L*) was small but significantly different between treatments (13.2 ± 2.7 and 8.2 ± 2.4 mm in well-watered and water-limited treatments, respectively; Supplementary Tables S1 and S2).

## Discussion

### Leaf carbon isotopic composition

Water-limited C_4_ plants consistently have lower δ^13^C_BL_ values than well-watered plants (Henderson *et al.*, 1998; Ghannoum *et al.*, 2002). Water limitations also typically reduce *g*_s_, and in C_4_ plants low *g*_s_ results in depleted δ^13^C_BL_. This is primarily because Δ^13^C_BL_ decreases with *C*_i_/*C*_a_ when ϕ is generally below 0.37, and low *g*_s_ tends to decrease *C*_i_/*C*_a_ (Cernusak *et al.*, 2013; von Caemmerer *et al.*, 2014). Alternatively, a decreased photosynthetic capacity in the water-limited plants could increase *C*_i_/*C*_a_, which would decrease Δ^13^C_BL_ and lead to an increase in δ^13^C_BL_ values. Therefore, the decrease in δ^13^C_BL_ observed in the water-limited plants is mostly due to changes in *g*_s_ and its influence on *C*_i_/*C*_a_. However, it is possible that water limitations increase ϕ, and lower δ^13^C_BL_ values.

Models of C_4_ isotope exchange suggest that a decrease in the capacity of the CO_2_ concentrating mechanism, as would occur with a stomatal limitation in CO_2_ supply, could decrease ϕ (Ellsworth and Cousins, 2016). Additionally, leaf level measurements of CO_2_ exchange have demonstrated that ϕ remains fairly constant under various environmental conditions (Henderson *et al.*, 1998; Ubierna *et al.*, 2011, 2013; Sun *et al.*, 2012; Cernusak *et al.*, 2013; Kromdijk *et al.*, 2014; von Caemmerer *et al.*, 2014). The ability of C_4_ plants to maintain and minimize ϕ in response to long-term changes in growth conditions is not surprising as the C_4_ and C_3_ cycles are metabolically coordinated between the mesophyll and bundle sheath cells, causing them to function as integrated and not independent cycles (Furbank *et al.*, 2013).

Using the simplified model of Δ^13^C (Eq. 2), the calculated ϕ from δ^13^C_BL_ and *C*_i_/*C*_a_ produced similar values (0.17–0.21) to what has been published previously (see list of studies in Kromdijk *et al.*, 2014). This difference in ϕ could account for a mean 22% and 24% of the measured difference in δ^13^C_BL_ across treatments in *S. italica* and *S. viridis*, respectively. In a separate study, under well-watered conditions Δ^13^C_instantaneous_ at similar *C*_i_/*C*_a_ was approximately 4.4 ± 0.2‰ for both *S. viridis* and *S. italica*, giving evidence that ϕ is not inherently different between these species (Ellsworth *et al.*, unpublished data). Additionally, the relationship between Δ^13^C_BL_ and *C*_i_/*C*_a_ was similar for both species under all treatments, potentially falling on the same line where ϕ controls the slope (see Fig 1; Ellsworth and Cousins, 2016). Granted these calculations of ϕ provide only an approximation because instantaneous measures of Δ^13^C and δ^13^C_BL_ are known to differ because of post-photosynthetic fractionations, as discussed below (von Caemmerer *et al.*, 2014; Ellsworth and Cousins, 2016).

### Post-photosynthetic fractionation

Post-photosynthetic fractionations of carbon compounds could influence δ^13^C_BL_; however, to influence δ^13^C_BL_ there must be a change in the leaf carbon mass balance by isotopic flux into or out of the leaf (von Caemmerer *et al.*, 2014). Potentially, water-limited plants differ in which carbon pools are exported from the leaf. For example, if enriched amino acids are transported from the leaf at a greater rate than in well-watered plants (Melzer and O’Leary, 1987), then δ^13^C_BL_ could decrease. However, enriched amino acids are a small pool of carbon compared with sucrose and cellulose, so their export would have to be extremely large to account for the observed depletion in δ^13^C_BL_. Additionally, the export or consumption by respiration of sucrose from starch degradation instead of from triose phosphate synthesis could also affect δ^13^C_BL_ because sucrose from starch degradation is more enriched in ^13^C (Hobbie and Werner, 2004; Tcherkez and Farquhar, 2005; von Caemmerer *et al.*, 2014). However, the respiratory carbon flux out of the leaf is relatively small compared with photosynthetic flux into the leaf, so water-limited plants would need an unrealistic shift in the isotopic signature of enriched respiratory carbon source (~12‰) to account for the −1‰ shift observed in δ^13^C_BL_. In water-limited C_3_ bean plants, sucrose was the primary carbon pool for respiration, suggesting that a dramatic shift in *Setaria* is unlikely (Duranceau *et al.*, 1999; Tcherkez *et al.*, 2003). Therefore, as discussed above, the shift in δ^13^C_BL_ is most likely not due to post-photosynthetic fractionations but rather to the treatment effect on leaf gas exchange and TE.

### Leaf carbon isotopic composition across drought experiments

In both species, δ^13^C_BL_ in water-limited plants consistently showed lower values by 0.9–1.1‰ than in the well-watered plants. Previous studies also have found a difference in δ^13^C_BL_ values between well-watered and water-limited plants of 0.2–0.6‰, which probably depended on the type, severity, or duration of the reduction in water availability (Saliendra *et al.*, 1996; Henderson *et al.*, 1998; Brück *et al.*, 2000; Monneveux *et al.*, 2007; Cabrera-Bosquet *et al.*, 2009*a*). Nonetheless, the consistent depletion in ^13^C in response to water limitations persisted across C_4_ species and experiments, lending further evidence that decreased *g*_s_ is driving the response in δ^13^C_BL_. Additionally, Gresset *et al.* (2014) found that δ^13^C_BL_ in C_4_ maize was under genetic control, giving support to the potential use of δ^13^C_BL_ as a genetic screen for TE. However, further research is needed to determine the degree to which δ^13^C_BL_ can be used to detect subtle differences in TE in C_4_ plants, if δ^13^C_BL_ is under similar genetic control as TE, and if it can be used to screen for TE across genotypes.

### Transpiration efficiency estimated from leaf carbon isotopic composition

Transpiration efficiency (TE_w_) calculated from δ^13^C_BL_ correlated strongly with leaf-level gas exchange measurements of *g*_s_ and TE_intrinsic_ (*A*_net_/*g*_s_). Therefore, calculating TE_w_ based on *C*_i_/*C*_a_ estimated from δ^13^C_BL_ accurately reflected differences in *g*_s_ between treatments. However, in *S. viridis*, TE_plant_ was more highly correlated with TE_intrinsic_ and TE_instantaneous_ than in *S. italica*. This may be because the short and bushy *S. viridis* has proportionally more leaf biomass than the upright *S. italica*, so leaf characteristics would have a greater influence on plant-level estimates of TE (e.g. TE_plant_) in *S. viridis* than in *S. italica*. Nonetheless, for both species, δ^13^C_BL_ reflected differences in both whole-plant and leaf-level estimates of TE.

### Leaf water enrichment

In *Setaria viridis*, Δ^18^O_LW_ formed a negative relationship with *E* as expected based on the Péclet model. In the Péclet model, the Péclet number is proportional to *E* and *L*, so a positive relationship between Δ^18^O_LW_ and *E* requires *L* to remain constant across individuals or treatments. *L* differed only slightly between treatments, and this did not remove the relationship between Δ^18^O_LW_ and *f* (1−Δ^18^O_LW_/Δ^18^O_e_) with *E*, showing evidence that the Péclet model best describes leaf water isotopic composition (Supplementary Fig. S1 and Fig. 4). The expected relationship existed for *S. viridis*, lending strength to the possible use of leaf oxygen isotopic composition as a proxy of *E*.

Contrary to *S. viridis*, Δ^18^O_LW_ and *E* did not form a significant relationship in *S. italica*, and Δ^18^O_LW_ values were more enriched than Δ^18^O_e_. One potential reason for this disparity in results between the two species is that leaf temperature differed between treatments and across the range of leaf temperature. High transpiration rate can change Δ^18^O_LW_ and subsequently Δ^18^O_BL_ by decreasing leaf temperature through evaporative cooling. Both ε^+^ and *e*_i_ (and therefore *e*_a_/*e*_i_) are temperature-dependent (Barbour, 2007; Barbour *et al.*, 2004). In the experiment with *S. italica*, the maximum difference in leaf temperature across the treatments was less than 2 °C (Ellsworth *et al.*, 2016); Δ^18^O_e_ of leaves of water-limited plants would only increase by ~0.6‰, and Δ^18^O_LW_, being partly composed of Δ^18^O_e_, would change by a fraction of 0.6‰. If leaf temperature in *S. viridis* differed less than 2 °C, as previously observed with *S. italica* (Ellsworth *et al.*, 2016), then the temperature difference between treatments would be insufficient to explain the difference in Δ^18^O_LW_ observed in *S. viridis*. As for *S. italica*, Δ^18^O_LW_ values showed the opposite trend as would be expected if the differences were based on leaf temperature. Therefore, the relationship between Δ^18^O_LW_ and *E* in *S. viridis* suggests a Péclet effect in *S. viridis* but not in *S. italica*.

A possible reason why *E* does not affect the mixing of source water with water from the sites of evaporation in *S. italica* (as described in the Péclet model and observed in *S. viridis*) may be because Δ^18^O_LW_ increases with leaf length in C_4_ grasses (Helliker and Ehleringer, 2000). In *S. italica*, longer leaf length in well-watered plants than in water-limited plants apparently increased Δ^18^O_LW_ values sufficiently to mask the expected relationship between Δ^18^O_LW_ and *E*. This effect of leaf length was strong enough that Δ^18^O_BL_ was positively correlated with *E* and *g*_s_. Enrichment up the leaf blade, described as the longitudinal Péclet effect, occurs because the xylem water being supplied to the sites of evaporation becomes progressively more enriched from the base to the tip of the leaf blade (Gan *et al.*, 2003). Therefore, relative to source water entering the leaf base, the water at the sites of evaporation is enriched above what can be attributed to *E*. In contrast, the treatment difference in leaf length in *S. viridis* was minimal and insufficient to mask the transpiration-derived differences in Δ^18^O_LW_.

### Bulk leaf enrichment and *E*

According to theory, bulk leaf enrichment (Δ^18^O_BL_) should reflect the isotopic signature of leaf water in which bulk tissue is synthesized (Farquhar and Lloyd, 1993; Sternberg *et al.*, 1986). As expected, a weak but significant positive relationship between Δ^18^O_LW_ and Δ^18^O_BL_ was found in *S. viridis*, but this relationship did not translate into a significant difference in Δ^18^O_BL_ between treatments or significant correlations of Δ^18^O_BL_ with measures of gas exchange or growth. Three possibilities exist that may explain this pattern. First, the relationship between Δ^18^O_LW_ and *E* primarily exists because of the longitudinal or xylem Péclet effect and not the mesophyll/lamina or radial Péclet effect. Therefore, the oxygen isotope signature of lamina water that is passed onto organic molecules may have little *E*-related enrichment, so the relationship between Δ^18^O_LW_ and *E* would not be passed onto the bulk leaf tissue (Holloway‐Phillips *et al.*, 2016). Second, Δ^18^O_LW_ was measured once, and a single measurement may not capture all variation in *E* and climatic conditions such as δ^18^O_v_, relative humidity, and leaf temperature over the leaf lifespan, which can be difficult to control or account for precisely even in a controlled environment setting (Roden and Siegwolf, 2012; Roden and Farquhar, 2012). Third, leaf length would not affect Δ^18^O_BL_ as much as Δ^18^O_LW_ because Δ^18^O_BL_ would be driven principally by Δ^18^O_LW_ early in leaf construction when oxygen isotope exchange between water and sucrose takes place. Nevertheless, the magnitude of this non-significant difference between treatments was similar to what has been reported in other studies (Cabrera‐Bosquet *et al.*, 2009*c*; Sánchez-Bragado *et al.*, 2016). The oxygen isotopic composition of other plant organs has been proposed as proxies because they produced stronger correlations with grain yield than δ^18^O_BL_ (Sánchez-Bragado *et al.*, 2016). However, it is necessary to understand how the leaf oxygen isotope enrichment that is related to *E* is passed onto these organs before their δ^18^O can be used an effective proxy for *E*.

Another problem that can obscure the relationship that Δ^18^O_LW_ and Δ^18^O_BL_ have with *E* is misidentifying source water (δ^18^O_S_) used to calculate Δ^18^O_LW_ and Δ^18^O_BL_. In this study, we measured the isotopic signature of three possible source waters: (i) irrigation water, (ii) mean soil water, and (iii) root crown water. Isotopically soil and root crown water were statistically indistinguishable, confirming previous studies that there is little fractionation of oxygen isotopes upon uptake by roots, so root crown water is a good representation of δ^18^O_S_ at the time of water collection (Ellsworth and Williams, 2007). Irrigation water does not reflect source water in water limitation studies because it undergoes evaporative enrichment, creating isotopically distinct soil water pools for each treatment. As a result, differences in Δ^18^O_LW_ and Δ^18^O_BL_ between treatments could simply be an artefact of incorrectly identifying δ^18^O_S_, and not because of other physiological traits or environmental factors. Therefore, care must be taken to define the real source water of leaves.

## Conclusion

Leaf δ^13^C had strong relationships with *E*, *g*_s_, water use, aboveground biomass production, and all measures of TE. Although the variation in δ^13^C_BL_ was less than that in C_3_ species, the overall consistency of the signal between well-watered and water-limited plants suggests that δ^13^C_BL_ may be an effective tool for distinguishing between well-watered and water-limited plants. However, more research is needed to determine if δ^13^C_BL_ can be used to detect differences in TE and *g*_s_ across more similar genotypes and serve as an effective proxy of TE in high throughput phenotyping across a range of field growth conditions. Alternatively, the use of Δ^18^O_BL_ as a proxy for transpiration rate in the C_4_ grass *Setaria* is problematic for three reasons. First, source water can be isotopically variable across time and different between treatment conditions, making accurate calculations of Δ^18^O_LW_ and Δ^18^O_BL_ difficult. Furthermore, assuming that both well-watered and water-limited plants have the same δ^18^O_S_ may lead to erroneous implications for differences in *E*. Second, either a small mesophyll Péclet effect where organic molecules are synthesized or leaf water oxygen exchange with lamina water in sucrose synthesis was not sufficient to pass the leaf water isotopic signature on to that of bulk leaf tissue, so that the subtle differences in Δ^18^O_BL_ across a gradient of *E* were weak. Finally, changes in leaf size in response to water limitations appeared to mask the expected relationship of Δ^18^O_LW_ and Δ^18^O_BL_ with *E*.

## Supplementary data

Supplementary data are available at *JXB* online.

Fig. S1. The relationship between proportional deviation of leaf water (Δ^18^O_LW_) from evaporative site water (Δ^18^O_e_) oxygen isotopic enrichment (*f*) and transpiration rate (*E*).

Table S1. *F* values, numerator degrees of freedom (ndf), denominator degrees of freedom (ddf) and *P* values from two-way ANOVA of the effects of a differential irrigation treatment and collection period on plant water use and growth, leaf water relations, and isotopic composition for *S. viridis*.

Table S2. Plant water relations, growth, and isotopic composition of *S. viridis* grown under well-watered and water-limited conditions and harvested during three collection periods.

Table S3. *F* values, numerator degrees of freedom (ndf), denominator degrees of freedom (ddf) and *P* values from one-way ANOVA of the effects of a differential irrigation treatment on plant water use and growth, leaf water relations, and isotopic composition for *S. italica*.

Table S4. Plant water relations, growth, and stable isotopes of *S. italica* grown under well-watered, moderately and severely water-limited treatments and harvested during two collection periods.

Table S5. Correlations between measured parameters of both well-watered and water-limited plants and leaf water enrichment (Δ^18^O_LW_), bulk leaf enrichment (Δ^18^O_BL_), and δ^13^C for *S. viridis*.

Table S6. Correlations of measured parameters with δ^13^C, Δ^18^O_LW_, and Δ^18^O_BL_ for *S. italica*.

## Supplementary Material

Supplementary Tables S1-S6 and Figure S1

## Appendix: Theory behind the relationship of δ^18^O_LW_, δ^18^O_BL_, and transpiration

Transpiration has been shown to affect the leaf water oxygen isotope composition (δ^18^O_LW_) in three different ways. The first two deal with the role that transpiration plays in modifying the evaporative environment under which leaf water becomes enriched. At the sites of evaporation near the stomates, the water undergoing evaporation becomes enriched in ^18^O relative to the source water entering the leaf via the xylem. The third way considers the effect that transpiration has on the quantitative contribution of enriched water at the sites of evaporation (δ^18^O_e_) to δ^18^O_LW_.

Leaf water isotopic composition (δ^18^O_LW_) can be considered a mixture of source water in the xylem (δ^18^O_S_) and water from the sites of evaporation and mesophyll (δ^18^O_e_):

$$\delta^{18}O_{\text{LW}}=f\times \delta^{18}O_{S}+\left(1-f\right)\delta^{18}O_{e}$$ (A1)

where *f* is the fraction of unenriched water in leaf veins. By expressing the oxygen isotopic composition in leaf water as enrichment above source water (Δ^18^O_LW_), any differences between in source water between leaves is accounted for and only changes in enrichment of ^18^O within the leaf are considered:

$$\Delta^{18}O_{\text{LW}}=\;\frac{\delta^{18}O_{\text{LW}}-\delta^{18}O_{S}}{1-\delta^{18}O_{\text{LW}}}$$ (A2)

Equation S1 can be expressed in terms of enrichments of ^18^O, where Δ^18^O_LW_ is equal to the proportional contribution of oxygen isotope enrichment in water from the sites of evaporation (Δ^18^O_e_) to Δ^18^O_LW_ (Barbour, 2007; Cernusak *et al.*, 2016):

$$\Delta^{18}O_{\text{LW}}=\;\left(1-f\right)\Delta^{18}O_{e}$$ (A3)

Considering Eq. A3, Δ^18^O_LW_ can vary across a gradient of *E* if either Δ^18^O_e_ or the proportional contribution of Δ^18^O_e_ (1−*f*) represented in the leaf changes with *E*.

The two ways in which Δ^18^O_e_ itself can be influenced by *E* are based on the effect that *E* has on leaf temperature and the resulting effect that changing leaf temperature has on the equilibrium fractionation (ε^+^) and intercellular air vapor mole fraction (*e*_i_) when *e*_a_ remains constant:

$$\Delta^{18}O_{e}=\varepsilon^{+}+\varepsilon_{k}+\left(\Delta^{18}O_{v}-\varepsilon_{k}\right)e_{a}/e_{i}$$ (A4)

This, in turn, influences Δ^18^O_LW_ (Eq S3). Δ^18^O_e_ is assumed to follow the Craig–Gordon model and can be derived from ε^+^, the ambient to intercellular air vapor mole fraction (*e*_a_/*e*_i_), the kinetic fractionation (ε_k_), and air vapor enrichment (Δ^18^O_v_) (Craig & Gordon, 1965; Dongmann et al., 1974; Flanagan *et al.*, 1991). Based on this equation, increasing *g*_s_ and subsequently *E* can either dry the intercellular area or increase ε^+^ by decreasing leaf temperature (*T*_L_), both of which increase Δ^18^O_e_. This because ε^+^ is inversely related to *T*_L_:

$$\varepsilon^{+}=2.644-3.206\left(\frac{10^{3}}{T_{L}}\right)+1.534\left(\frac{10^{6}}{T_{L}^{2}}\right)$$ (A5)

*E* can decrease leaf temperature by increasing evaporative cooling and consequently increase ε^+^ (Gates, 1968). Ascribing changes in Δ^18^O_e_ to variation in *E* assumes that Δ^18^O_v_ and *e*_a_ remain constant.

Contrastingly, *ε*_k_ is inversely proportional to the stomatal (*g*_s_) and boundary layer (*g*_bl_) conductances, so increasing *g*_s_ decreases ε_k_:

$$\varepsilon_{k}=\;\frac{32\;g_{s}^{-1\;}+21g_{\text{bl}}^{-1}}{g_{s}^{-1}+\;g_{\text{bl}}^{-1}}$$ (A6)

The third way in which *E* can change Δ^18^O_e_ and consequently Δ^18^O_LW_ is by modifying the contribution (1−*f* in Eq. A3) of Δ^18^O_e_ to Δ^18^O_LW_. The Péclet model was developed to explain how Δ^18^O_LW_ values were often lower than Δ^18^O_e_ values, meaning that something is influencing Δ^18^O_LW_ other than the evaporative enrichment (Farquhar et al., 1998). It describes the extent to which the advection of relatively unenriched xylem water mixes with enriched water from the sites of evaporation. This depends on the Péclet number (ρ), which is proportional to *E* and the effective path length (*L*) that water takes to move from the veins to the sites of evaporation, and inversely proportional to the molar density of water (*C*) and the diffusivity of H_2_^18^O in water (*D*) (Farquhar et al., 1998; Barbour & Farquhar, 2000; Barbour et al., 2000*b*; Gan *et al.*, 2003; Barbour & Farquhar, 2004; Barbour, 2007):

$$\rho =\frac{LE}{CD}$$ (A7)

ρ is then related to Δ^18^O_LW_ according to:

$$\Delta^{18}O_{\text{LW}}=\;\frac{\Delta^{18}O_{e}\;\left(1-e^{-\rho }\right)}{\rho }$$ (A8)

If *L* remains constant, then ρ increases with *E*, meaning that back diffusion of water from sites of evaporation decreases relative to the advection of unenriched water from the xylem to the sites of evaporation. Simply stated, the relative contribution of Δ^18^O_e_ to Δ^18^O_LW_ decreases, and Δ^18^O_LW_ decreases with increasing *E*, assuming that *L* remains constant.

Bulk leaf enrichment above source water (Δ^18^O_BL_) reflects both Δ^18^O_LW_, the biochemical fractionation during the synthesis of organic compounds, and the degree of oxygen exchange during synthesis of cellulose (Craig & Gordon, 1965; Dongmann *et al.*, 1974; Yakir, 1992; Barbour, 2007):

$$\Delta^{18}O_{\text{BL}}=\;\Delta^{18}O_{\text{LW}}\left(1-p_{\text{ex}}p_{x}\right)+\;\varepsilon_{\text{wc}}+\;\varepsilon_{\text{cp}}$$ (A9)

Where *p*_ex_ is the proportion of exchangeable oxygen in cellulose being formed from glucose, *p*_x_ is the proportion of source water at the site of cellulose/tissue synthesis, ε_wc_ is the biochemical fractionation factor during the carbonyl oxygen exchange with water during cellulose synthesis, and ε_cp_ is the isotopic difference between cellulose and whole tissue. This equation assumes a constant ε_cp_, although this factor may vary among leaves and with leaf age Cernusak et al., 2005. Therefore, in the scenario where Δ^18^O_LW_ varies across a gradient of *E*, Δ^18^O_BL_ integrates this variation over the period of leaf construction. In this case, Δ^18^O_BL_ may be used as a proxy for *E* during leaf growth (Barbour *et al.*, 2000a; Barbour, 2007).

The concept behind the Péclet effect theory that the proportional deviation of Δ^18^O_LW_ from Δ^18^O_e_ (*f* in Eq. A3) increases along a gradient of *E* has failed to be observed in several studies (Roden & Ehleringer, 1999; Cernusak et al., 2003; Loucos et al., 2015; Song et al., 2015; Holloway‐Phillips et al., 2016). The failure to find this relationship in many instances has been explained by changes in factors such as *T*_L_ and *e*_i_ with *E*, as discussed above and (Barbour et al., 2000a,b, 2004; Barbour & Farquhar, 2004). Song *et al.* (2015) found that the two-pool model proposed by Roden and Ehleringer (1999) better described the response in Δ^18^O_LW_ to environmental conditions in upland cotton (*Gossypium hirsutum*) than the Péclet model. Additionally, the assumption that *L* is constant for species and across a gradient of transpiration rates has not always been supported (Song et al., 2013; Roden *et al.*, 2015). If *L* does not remain constant across a range of *E*, then it obscures any relationship that may exist between *E* and Δ^18^O_LW_ or Δ^18^O_BL_.

Vein density and vein volume fraction affect the enrichment of leaf water because of the strong Péclet effect within the xylem (Helliker & Ehleringer, 2000; Farquhar & Gan, 2003; Gan et al., 2003; Holloway‐Phillips et al., 2016). This Péclet has been called the longitudinal or xylem Péclet and is greater in magnitude than the radial or mesophyll Péclet (Farquhar & Gan, 2003; Gan et al., 2003; Holloway‐Phillips et al., 2016). Vein volume fraction increases the relative importance of xylem water in Δ^18^O_LW_, which makes the influence of the xylem Péclet increasingly measureable in Δ^18^O_LW_. This means that the xylem and not the mesophyll may be the location of the Péclet effect (Holloway‐Phillips *et al.*, 2016). Since the mesophyll is where sugars are synthesized, this may explain why the oxygen isotope signature of leaf water is not always reflected in bulk leaf tissue.
