# Supplementary material for: Relationship of leaf oxygen and carbon isotopic composition with transpiration efficiency in the C4 grasses Setaria viridis and Setaria italica
Source: J Exp Bot. 2017 Jul 11;68(13):3513–28. doi: 10.1093/jxb/erx185 (PMC5853516; doi:10.1093/jxb/erx185)
Supplement: Supplementary Tables S1-S6 and Figure S1 [file erx185_suppl_supplementary_tables_s1_s6_figure_s1.pdf]

Supplementary Data

Table S1. *F* values, numerator degrees of freedom (ndf), denominator degrees of freedom (ddf) and *P* values from two-way ANOVA of the effects of a differential irrigation treatment and collection period on plant water use and growth, leaf water relations, and isotopic composition for *S. viridis*. Levels of significance were calculated from 2-way ANOVAs described in the 'Materials and Methods' (P > 0.05).

	Differential irrigation		Collection time		Differential irrigation x Collection time	
Measurements	$F_{ndf,ddf}$	*P*	$F_{ndf,ddf}$	*P*	$F_{ndf,ddf}$	*P*
Specific leaf area (SLA; m^2 kg^{-1} leaf)	**$13.107_{1,21}$**	**< 0.01**	$0.739_{2,21}$	0.49	$1.822_{2,21}$	0.19
Total water use (L)	**$135.227_{1,24}$**	**< 0.001**	**$23.730_{2,24}$**	**< 0.001**	$0.427_{2,24}$	0.66
Fresh aboveground biomass (g)	**$81.822_{1,24}$**	**< 0.001**	**$7.808_{2,24}$**	**0.002**	$2.070_{2,24}$	0.15
Dry aboveground biomass (g)	**$129.011_{1,24}$**	**< 0.001**	**$22.171_{2,24}$**	**< 0.001**	$0.298_{2,24}$	0.75
Number of tillers	**$37.230_{1,23}$**	**< 0.001**	$1.787_{2,23}$	0.19	$0.231_{2,23}$	0.80
$\Delta^{18}O_{LW}$	**$44.387_{1,23}$**	**< 0.001**	**$52.124_{2,23}$**	**< 0.001**	$1.971_{2,23}$	0.16
$\Delta^{18}O_{BL}$	**$9.761_{1,23}$**	**0.005**	**$3.945_{2,23}$**	**0.03**	$0.555_{2,23}$	0.58
$\delta^{13}C_{BL}$	**$53.055_{1,24}$**	**< 0.001**	$1.684_{2,24}$	0.21	$0.454_{2,24}$	0.64
$\delta^{18}O_{top\ soil}$	**$4.811_{1, 23}$**	**0.04**	$1.131_{2, 23}$	0.34	$0.339_{2, 23}$	0.72
$\delta^{18}O_{root\ crown}$	**$66.005_{1,23}$**	**< 0.001**	**$6.421_{2,23}$**	**< 0.01**	$2.047_{2,23}$	0.15
C_i/C_a	**$50.877_{1,23}$**	**<0.001**	**$3.651_{2,23}$**	**0.04**	$0.444_{2,23}$	0.44
g_s (mmol H_2O m^{-2} s^{-1})	**$46.209_{1,23}$**	**< 0.001**	**$5.236_{2,23}$**	**0.01**	$3.557_{2,23}$	0.05
E (mmol H_2O m^{-2} s^{-1})	**$73.172_{1,23}$**	**< 0.001**	$2.839_{2,23}$	0.08	**$16.068_{2,23}$**	**< 0.001**
A_{net} (µmol CO_2 $m^{-2}s^{-1}$)	**$9.028_{1,23}$**	**0.006**	**$4.137_{2,23}$**	**0.03**	**$21.725_{2,23}$**	**< 0.001**
$TE_{instantaneous}$ (A_{net}/E)	**$28.790_{1,23}$**	**< 0.001**	**$6.921_{2,23}$**	**0.004**	$0.747_{2,23}$	0.49
$TE_{intrinsic}$ (A_{net}/g_s)	**$84.648_{1,23}$**	**< 0.001**	**$7.943_{2,24}$**	**0.002**	$2.017_{2,24}$	0.16
$TE_{long\ term}$ (g biomass L H_2O^{-1})	**$292.11_{1,24}$**	**< 0.001**	**$7.748_{2,24}$**	**0.003**	$0.755_{2,24}$	0.48
TE_W (derived from $\delta^{13}C_{BL}$)	**$236.169_{1,24}$**	**< 0.001**	$1.493_{2,24}$	0.25	$0.216_{2,24}$	0.81
Leaf length (cm)	**$35.498_{1,23}$**	**< 0.001**	$2.943_{2,23}$	0.07	$0.016_{2,23}$	0.98
Leaf water per area (L H_2O m^{-2} leaf)	$2.484_{1,21}$	0.13	**$16.482_{2,21}$**	**< 0.001**	$0.278_{2,21}$	0.76
Leaf relative water content (%)	**$544.22_{1,22}$**	**< 0.001**	**$113.68_{2,22}$**	**< 0.001**	**$31.74_{2,22}$**	**< 0.001**
Final number of panicles	**$34.597_{1,23}$**	**< 0.001**	**$7.662_{2,23}$**	**< 0.001**	$0.887_{2,23}$	0.43
Final ratio of panicles: tillers	$1.081_{1,23}$	0.31	**$6.259_{2,23}$**	**< 0.01**	$2.327_{2,23}$	0.12
Effective path length (mm)	**$6.614_{1,23}$**	**0.02**	**$8.425_{2,23}$**	**< 0.01**	$0.545_{2,23}$	0.59

Table S2. Plant water relations, growth, and isotopic composition of *S. viridis* grown under well-watered and water-limited conditions and harvested during three collection periods. Measurements of water use were made at both the plant and leaf level. The well-watered treatment was watered nightly to pot capacity. The plants in the water-limited treatment were given enough water daily to maintain a minimum GWC of 1.0. Means ± S.E. and level of significance were calculated for each parameter as described in 'Materials and methods'. Within the same row, means followed by the same letter are not significantly different ($P > 0.05$). Each variable was analyzed with a separate two-factor ANOVA (factors: collection periods and treatment). For initial panicle to tiller ratio, $\delta^{18}O_{topsoil}$, SLA, only the treatment main effect was significantly different with the water-limited treatment being greater except in SLA. For final panicle to tiller ratios, only collection time was significantly different with the ratio increasing with collection.

**Parameters**	**Collection 1**		**Collection 2**		**Collection 3**	
	**Well-watered**	**Water-limited**	**Well-watered**	**Water-limited**	**Well-watered**	**Water-limited**
Number of panicles	52 ± 2^{bc}	41 ± 4^{c}	84 ± 6^{ab}	47 ± 3^{c}	96 ± 10^{a}	48 ± 4^{c}
Panicles: tillers	0.42 ± 0.02	0.56 ± 0.06	0.58 ± 0.05	0.61 ± 0.05	0.68 ± 0.04	0.62 ± 0.06
$\delta^{18}O_{root\ crown}$	-16.7 ± 0.3^{c}	-14.9 ± 0.2^{a}	-16.5 ± 0.2^{bc}	-15.5 ± 0.2^{ab}	-15.9 ± 0.1^{bc}	-14.7 ± 0.2^{a}
SLA ($m^2\ kg^{-1}$ leaf)	258 ± 11.2	247.5 ± 5.6	256.7 ± 12.8	227.2 ± 3.3	266.3 ± 11.8	219.9 ± 11.7
Leaf relative water content (leaf water)	0.84 ± 0.002^{a}	0.78 ± 0.003^{c}	0.82 ± 0.006^{b}	0.76 ± 0.002^{d}	0.81 ± 0.003^{b}	0.7 ± 0.005^{e}
Water per leaf area (L H_2O m dry leaf^{-2})	0.14 ± 0.01^{a}	0.13 ± 0.01^{ab}	0.12 ± 0.003^{abc}	0.11 ± 0.002^{bc}	0.11 ± 0.001^{bc}	0.1 ± 0.01^{c}

Table S3. *F* values, numerator degrees of freedom (ndf), denominator degrees of freedom (ddf) and *P* values from one-way ANOVA of the effects of a differential irrigation treatment on plant water use and growth, leaf water relations, and isotopic composition for *S. italica*. Levels of significance were calculated from the one-way ANOVAs as described in the 'Materials and Methods'.

Measurements	$F_{ndf, ddf}$	*P*
Total water use (L)	**$62.45_{2, 29}$**	**< 0.001**
Fresh aboveground biomass (g)	**$53.34_{2, 29}$**	**< 0.001**
Dry aboveground biomass (g)	**$47.02_{2, 29}$**	**< 0.001**
Number of tillers	$1.143_{2, 29}$	0.33
$\Delta^{18}O_{LW}$	$0.181_{2, 29}$	0.84
$\Delta^{18}O_{BL}$	**$4.935_{2, 29}$**	**0.01**
$\delta^{13}C_{BL}$	**$28.34_{2, 29}$**	**< 0.001**
$\delta^{18}O_{top\ soil}$	**$33.45_{2, 29}$**	**< 0.001**
g_s (mmol^{-1} H_2O m^{-2} s^{-1})	**$21.22_{2, 29}$**	**< 0.001**
E (mmol^{-1} H_2O m^{-2} s^{-1})	**$46.99_{2, 29}$**	**< 0.001**
A_{net} (μmol^{-1} CO_2 m^{-2} s^{-1})	**$10.82_{2, 29}$**	**< 0.001**
$TE_{instantaneous}$ (A_{net}/E)	**$22.32_{2, 29}$**	**< 0.001**
$TE_{intrinsic}$ (A_{net}/g_s)	**$43.4_{2, 29}$**	**< 0.001**
$TE_{long\ term}$ (g dry biomass L H_2O transpired^{-1})	**$5.074_{2, 29}$**	**0.01**
TE_W (calculated from C_i/C_a derived from $\delta^{13}C_{BL}$)	**$28.37_{2, 29}$**	**< 0.001**
Leaf length (cm)	**$30.95_{2, 29}$**	**< 0.0001**
Fresh leaf weight (g)	**$26.000_{2, 29}$**	**< 0.001**
Fresh stem weight (g)	**$50.18_{2, 29}$**	**< 0.001**
Fresh panicle weight (g)	**$10.26_{2, 29}$**	**< 0.001**
Specific leaf area (SLA; m^2 kg^{-1} leaf)	**$9.911_{2, 28}$**	**< 0.001**
Leaf water per area (L H_2O m dry leaf^{-2})	**$4.276_{2, 28}$**	**0.02**
Leaf relative water content (%)	$0.153_{2, 28}$	0.86
Dry leaf weight (g)	**$30.75_{2, 29}$**	**< 0.001**
Dry stem weight (g)	**$32.36_{2, 29}$**	**< 0.001**
Dry panicle weight (g)	**$9.147_{2, 29}$**	**< 0.001**
Final number of panicles	**$5.235_{2, 29}$**	**0.01**
Number of large tillers	**$18.26_{2, 29}$**	**< 0.001**
Number of medium tillers	$1.923_{2, 29}$	0.79
Number of small tillers	$0.822_{2, 29}$	0.45
Number of large panicles	**$18.86_{2, 29}$**	**< 0.001**
Number of medium panicles	$1.635_{2, 29}$	0.21
Number of small panicles	$1.832_{2, 29}$	0.19
Leaf water enrichment ($\Delta^{18}O_{LW}$) based on root crown	$0.222_{2, 29}$	0.80
$\delta^{18}O_{top\ soil}$	**$33.45_{2, 29}$**	**< 0.001**
$\delta^{18}O_{root\ crown}$	**$38.25_{2, 29}$**	**< 0.001**

Table S4. Plant water relations, growth, and stable isotopes of *S. italica* grown under well-watered, moderately- and severely water-limited treatments and harvested during two collection periods. The chamber conditions for gas exchange measurements were 900 µmol m^{-1} s^{-1} PAR, leaf temperature of 29°C, flow rate of 300 m^{-2} s^{-1}, 21 % O_2, 380 µmol mol^{-1} CO_2. Means ± S.E. were calculated for each parameter as described in 'Materials and methods'. Within the same row, means followed by the same letter are not significantly different (P > 0.05).

Variables	Well-watered	Moderate water limitations	Severe water limitations
TE_W (based on measured C_i/C_a)	3.67 ± 0.35[b]	6.03 ± 0.33[a]	5.88 ± 0.31[a]
Fresh leaf mass (g)	174.4 ± 14.6[a]	52.4 ± 5.1[b]	40.0 ± 2.4[b]
Fresh stem mass (g)	249.5 ± 16.6[a]	76.5 ± 3.9[b]	53.5 ± 1.7[c]
Fresh panicle mass (g)	62.2 ± 13.7[a]	11.8 ± 2.0[b]	6.9 ± 1.1[b]
Dry leaf mass	39.5 ± 3.6[a]	13.7 ± 1.1[b]	10.4 ± 0.5[c]
Dry stem mass	50.4 ± 3.7[a]	16.6 ± 0.9[b]	12.7 ± 0.8[c]
Dry panicle mass	14.8 ± 3.4[a]	3.2 ± 0.6[b]	1.9 ± 0.3[b]
Final number of panicles	6.8 ± 1.5[a]	2.9 ± 0.3[ab]	2.2 ± 0.6[b]
Final ratio of panicles: Tillers	0.78 ± 0.10[a]	0.51 ± 0.07[ab]	0.28 ± 0.07[b]
Number of large tillers	5.00 ± 0.68[a]	3.27 ± 0.19[b]	1.82 ± 0.23[c]
Number of medium tillers	3.1 ± 0.74	2.36 ± 0.66	4.27 ± 0.71
Number of small tillers	1.10 ± 0.69	1.00 ± 0.36	1.82 ± 0.42
Number of large panicles	2.4 ± 0.50[a]	0.27 ± 0.14[b]	0.18 ± 0.12[b]
Number of medium panicles	1.6 ± 0.45	1.09 ± 0.21	0.73 ± 0.19
Number of small panicles	2.8 ± 0.85	1.55 ± 0.31	1.27 ± 0.54
$\delta^{18}O_{root\ crown}$	-15.9 ± 0.1[c]	-13.9 ± 0.2[b]	-12.7 ± 0.4[a]
$\Delta^{18}O_{LW}$ (based on root crown as source water)	21.18 ± 0.81	20.42 ± 1.15	20.24 ± 1.11
SLA (m^2 kg^{-1} leaf)	13.23 ± 0.96[b]	15.31 ± 0.48[ab]	18.17 ± 1.29[a]
Leaf relative water content (%)	collection 1 (0.73 ± 0.01) [a] collection 2 (0.70 ± 0.01) [b]		
Water per leaf area (L water m dry leaf^{-2})	0.19 ± 0.008[a]	0.17 ± 0.013[ab]	0.15 ± 0.009[b]

Table S5. Correlations between measured parameters of both well-watered and water-limited plants and leaf water enrichment ($\Delta^{18}O_{LW}$), bulk leaf enrichment ($\Delta^{18}O_{BL}$), and $\delta^{13}C$ for *S. viridis*. Both $\Delta^{18}O_{LW}$ and $\Delta^{18}O_{BL}$ were calculated using root crown water ($\delta^{18}O_{root\ crown}$) as the water source. Significant correlations are in bold, and the level of significance is given. Levels of significance are * $P < 0.05$, ** $P < 0.01$, *** $P < 0.001$, ns not significant ($P > 0.05$).

**Parameter**	$\mathbf{\Delta^{18}O_{LW}}$	$\mathbf{\Delta^{18}O_{BL}}$	$\mathbf{\delta^{13}C}$
Number of tillers	-0.30	-0.23	**0.76*****
Number of panicles	-0.06	-0.04	**0.57****
Panicles:tillers ratio	0.33	0.10	-0.14
Relative water content (%)	**-0.70*****	**-0.45***	**0.56****
Specific leaf area (SLA)	-0.32	-0.35	**0.63*****
Effective path length (L)	**-0.82*****	**-0.57****	**0.44***

Table S6. Correlations of measured parameters with $\delta^{13}C$, $\Delta^{18}O_{LW}$, and $\Delta^{18}O_{BL}$ for *S. italica*. Significant correlations (r) are in bold, and the level of significance is given. Levels of significance are * P < 0.05, ** P < 0.01, *** P < 0.001.

**Parameter**	$\delta^{13}C$	$\Delta^{18}O_{LW}$	$\Delta^{18}O_{BL}$
Number of tillers	0.22	0.04	-0.04
Number of panicles	**0.48****	0.22	0.23
Fresh leaf mass	**0.75*****	0.10	**0.43***
Fresh stem mass	**0.77*****	0.05	**0.42***
Fresh panicle mass	**0.66*****	0.14	0.24
Dry leaf biomass	**0.71*****	0.10	**0.47****
Dry stem biomass	**0.76*****	0.16	**0.44***
Dry panicle mass	**0.62*****	0.15	0.23
Relative leaf water content	-0.02	**-0.50****	**0.39***
Specific leaf area	-0.22	-0.23	**-0.45****
Leaf length	**0.68*****	0.00	0.28

Figure S1. The relationship between proportional deviation of leaf water ($\Delta^{18}O_{LW}$) from evaporative site water ($\Delta^{18}O_e$) oxygen isotopic enrichment (*f*) and transpiration rate (*E*). *S. viridis* showed a significant relationship as expected in the Péclet model ($f = 0.086\ E - 0.221$, $R^2 = 0.36$, $P = 0.0005$), but in *S. italica* no relationship was present. Note that the $\Delta^{18}O_{LW}$ values were greater than the predicted $\Delta^{18}O_e$ from the Craig-Gordon model, while in *S. viridis* $\Delta^{18}O_e$ was mostly greater than $\Delta^{18}O_{LW}$.

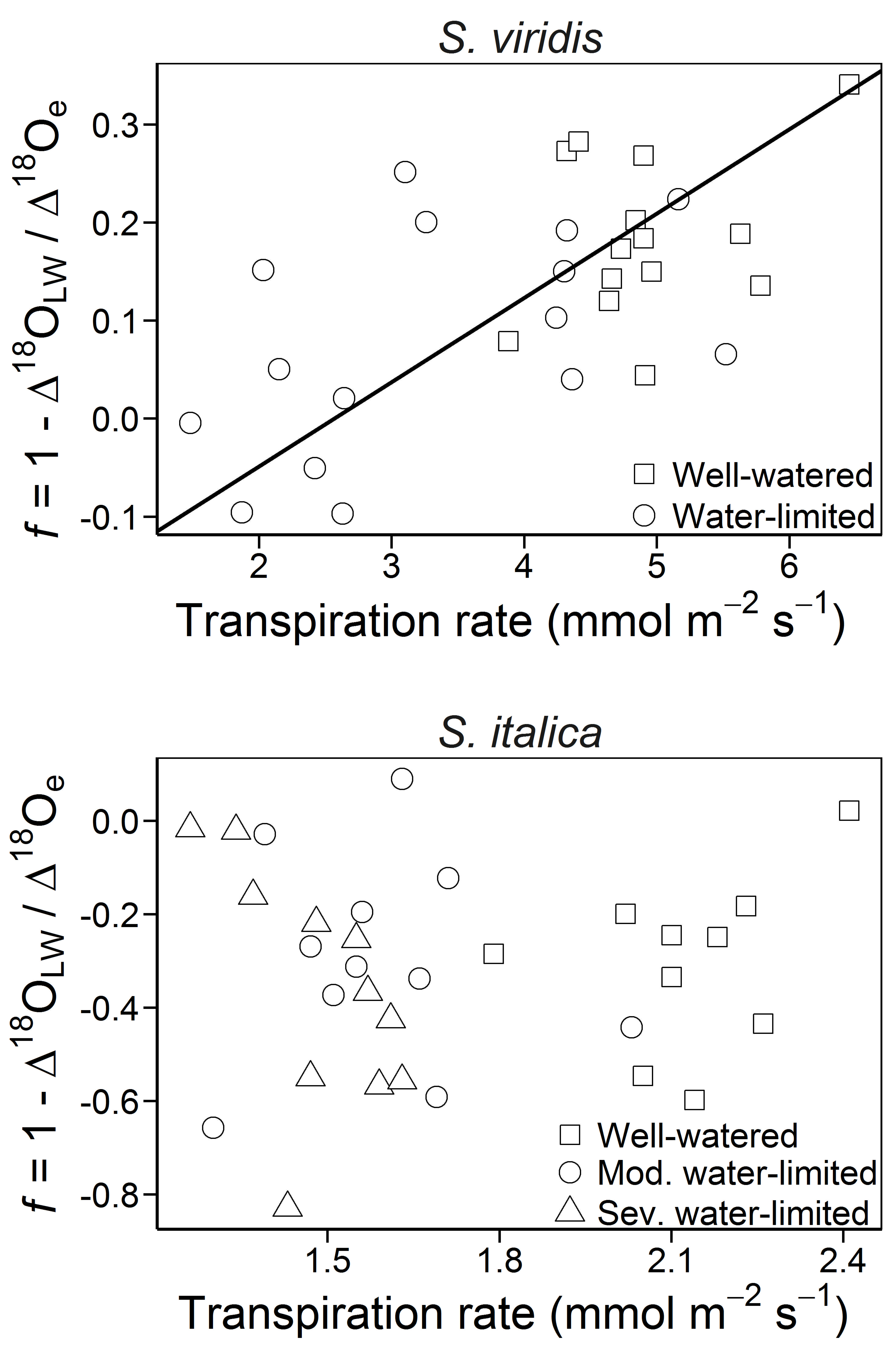

S. viridis
f = 1 - Δ18OLW / Δ18Oe
0.3
0.2
0.1
0.0
-0.1
2
3
4
5
6
Transpiration rate (mmol m−2 s−1)
Well-watered
Water-limited
S. italica
f = 1 - Δ18OLW / Δ18Oe
0.0
-0.2
-0.4
-0.6
-0.8
1.5
1.8
2.1
2.4
Transpiration rate (mmol m−2 s−1)
Well-watered
Mod. water-limited
Sev. water-limited
